# Microscale Mapping of Fiber Strain and Damage in Composite Wrinkled Laminates Using Computed Tomography Assisted Wide‐Angle X‐Ray Scattering

**DOI:** 10.1002/advs.202519053

**Published:** 2025-12-03

**Authors:** Hoang Minh Luong, James Trevarthen, Igor Dolbnya, Wadwan Singhapong, Nicholas Pilato, Richard Butler, Jiraphant Srisuriyachot, Alexander J. G. Lunt

**Affiliations:** ^1^ Centre for Integrated Materials, Processes & Structures (IMPS) Department of Mechanical Engineering University of Bath Bath BA2 7AY UK; ^2^ Dowty Propellers – GE Aviation Cheltenham GL2 9QH UK; ^3^ Diamond House Harwell Science and Innovation Campus Fermi Ave Didcot OX11 0DE UK

**Keywords:** carbon fibers reinforced polymer (CFRP) composites, lattice strain mapping, out‐of‐plane wrinkles, wide angle X‐ray scattering (WAXS), X‐ray computed tomography (XCT)

## Abstract

Out‐of‐plane fiber wrinkles in carbon‐fiber‐reinforced polymer laminates trigger premature failure, yet remain difficult to detect and assess. This study introduces a powerful new diagnostic capability: the pairing of X‐ray computed tomography (XCT) and Wide Angle X‐ray Scattering (WAXS) during in situ compression of specimens containing small (0.2 mm) and large (0.5 mm) wrinkles. This approach enables, for the first time, detailed field‐resolved mapping of axial ($\varepsilon_{100}$) and radial ($\varepsilon_{002}$) lattice microstrain. A new orientation‐aware reduction pipeline supports texture classification, peak fitting, and per‐point zero‐load referencing, requiring minimal intervention and enabling scalable industrial deployment. In large wrinkles, radial microstrain reached −14.5 µεMPa ^−1^, compared to −11.0 µεMPa ^−1^ axially; small wrinkles exhibit approximately one‐third of this magnitude. Strain hotspots are identified prior to failure, and tomography confirms these regions as the origin of delamination, matrix cracking, and fiber kink banding. To verify the results analytically, a compact, orientation‐aware predictor is developed, reproducing measured fields with a mean absolute error on the order of $10^{-3}$. These findings establish radial microstrain gradients as a robust, non‐destructive indicator of wrinkle severity, providing unique insight and enabling defect behavior to be embedded into full‐scale modeling. This supports performance‐based rejection criteria and targets inspection in aerospace laminates.

## Graphical abstract

This study combines full‐field tomography with diffraction mapping to quantify radial ($\varepsilon_{002}$) and axial ($\varepsilon_{100}$) lattice strain in wrinkled carbon‐fiber specimens for the first time. Radial microstrain gradients (−14.5 µεMPa ^−1^) are found to signal damage‐prone zones ahead of failure, which is cross‐validated via tomography. The approach supports predictive modeling and performance‐based inspection, offering a scalable route for aerospace‐grade composite triage.

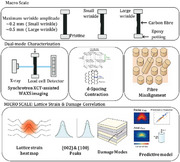

## 1 Introduction

Carbon‐fibers reinforced polymer (CFRP) composites are widely employed in aerospace applications due to their high specific strength and fatigue resistance.^[^ ^1^ ^]^ For example, modern propeller blades use hybrid carbon/glass‐fiber laminates with unidirectional plies to achieve an optimal balance of stiffness, toughness and weight.^[^ ^2^, ^3^ ^]^ However, the structural integrity of such composites is susceptible to manufacturing defects. In particular, out‐of‐plane fiber wrinkles are a common defect in complex laminate components that are associated with geometric deviations of fiber layers from the intended lay.^[^ ^4^ ^]^ These wrinkles act as potent failure‐initiation sites, significantly degrading mechanical performance by reducing tensile, compressive, flexural and fatigue strength.^[^ ^5^, ^6^, ^7^, ^8^ ^]^ Even relatively small wrinkles can precipitate premature failure under load, so detecting wrinkles and the associated early‐stage damage is of paramount importance. Therefore, ensuring effective understanding of the influence of wrinkles is critical for numerous safety‐critical composite parts in aviation and wind‐energy structures.^[^ ^8^, ^9^, ^10^ ^]^ From a fundamental standpoint, wrinkles present an opportunity to investigate how local fiber misalignments translate to stress concentrations and damage evolution in a multi‐material system, providing insight that can improve design guidelines and manufacturing processes.^[^ ^8^, ^11^ ^]^

Despite their importance, wrinkle defects are challenging to detect and characterise, especially in the early stages of loading before failure is initiated. This is because wrinkles are typically embedded deep within laminated components, meaning that surface‐based inspections can yield inconclusive results.^[^ ^12^ ^]^ However, active techniques such as in‐situ 3‐D digital image correlation under low tensile load^[^ ^11^ ^]^ or vacuum/thermal shearography that images strain while the structure is loaded,^[^ ^13^ ^]^ have proven capable of revealing subsurface fiber wrinkles long before catastrophic failure.

Additionally, a variety of non‐destructive evaluation (NDE) techniques have been explored to locate and assess wrinkles in composites.^[^ ^14^ ^]^ Traditional ultrasonic scanning is the dominant industrial method for subsurface‐defect detection (including delaminations and barely‐visible impact damage) and this approach has been adapted for wrinkle detection.^[^ ^15^ ^]^ Ultrasonic methods, however, face challenges: because wrinkles do not fully sever material continuity and they generate only weak reflection signals, so advanced signal processing or phased‐array techniques are required to resolve them.^[^ ^16^ ^]^ Thermography and eddy‐current scanning have also been attempted, but these are limited to near‐surface regions or demand specific loading conditions to accentuate wrinkle‐induced stress fields.^[^ ^10^ ^]^

X‐ray imaging methods, particularly X‐ray micro‐computed tomography (micro‐CT), can directly capture the 3D geometry of wrinkles and neighboring defects.^[^ ^17^ ^]^ For example, laboratory micro‐CT has been used to quantify fiber waviness in millimetre‐scale composite coupons.^[^ ^18^, ^19^ ^]^ Micro‐CT has also been employed to visualise internal flaws such as fiber breaks and delamination in small coupons.^[^ ^20^, ^21^ ^]^ However, conventional micro‐CT is constrained by spatial resolution, the low X‐ray contrast between carbon fibers and polymer, and sample‐size limitations.^[^ ^14^, ^22^ ^]^ Consequently, inspecting large, thick aerospace components via micro‐CT is often impractical, and even in small samples, the technique may miss very early‐stage damage (e.g. hairline matrix cracks) when resolution or contrast is insufficient. In summary, existing single‐modality NDE approaches either provide detailed geometric information or indirect indications of internal stress, but rarely both. This gap hinders understanding of how wrinkle damage is initiated as they are unable to simultaneously reveal the evolution of physical damage and the material state (strain, fibers alignment) that drives this damage.^[^ ^23^, ^24^, ^25^ ^]^

Advanced synchrotron X‐ray techniques offer a promising route to fill this gap. Synchrotron X‐ray computed tomography (XCT) can achieve high spatial resolution (on the order of a few micrometres or better) with short exposure times, enabling in‐depth observation of microscale damage in composites.^[^ ^26^, ^27^ ^]^ In contrast to laboratory micro‐CT, synchrotron XCT offers superior image quality and the potential for phase contrast, which is particularly advantageous for low‐contrast materials such as carbon/epoxy.^[^ ^28^ ^]^ Previous in‐situ synchrotron X‐ray computed‐tomography investigations have visualized, in three dimensions and with scan times from 1 s to a few minutes, the evolution of matrix cracking, fibers/matrix debonding, ply‐level delamination and fibers breakage in carbon‐fiber‐reinforced polymers.^[^ ^26^, ^29^ ^]^ For instance, in situ tomography during mechanical tests has revealed how cracks initiate at defects or how fiber breaks accumulate, information unattainable from surface measurements.^[^ ^30^, ^31^ ^]^ Nevertheless, while XCT pinpoints where and what damage occurs, it does not directly measure the internal stresses or strains that lead to such damage.^[^ ^32^ ^]^

Conversely, high‐energy Wide Angle X‐ray Scattering (WAXS) techniques can probe the internal lattice structure of composite constituents, offering insight into the state of stress and fiber orientation within the material.^[^ ^33^ ^]^ Throughout this paper, the radial (inter‐layer) fiber strain derived from {002} is denoted $\varepsilon_{002}$, and the axial (basal‐plane‐parallel) fiber strain from {100} is denoted $\varepsilon_{100}$; “radial/axial” refer to directions normal/parallel to the fiber axis, respectively. It is important to note that the radial strain here refers only to the radial strain within the fibers in a direction perpendicular to the incident beam, in other words the in‐plane radial strains within the heat maps presented below. This is related to the principle of WAXS in which scattering does not occur from planes with a Miller index parallel to the incident beam.

WAXS is a well‐established tool for mapping strain fields in crystalline materials such as metals or ceramics; its application to polymer composites, which contain partially crystalline carbon fibers, is more recent. Carbon fiber possess a turbostratic (graphitic) microstructure that produces distinct diffraction peaks whose positions and widths can be analysed to infer elastic strain and preferred orientation of the fibers.^[^ ^14^, ^33^, ^34^, ^35^ ^]^ One important distinction here is that WAXS measures only atomic‐scale lattice strain within a particular phase (in this case, the fiber) which is related to, but distinct from the average strain response of the component, which can contain other phases (such as the epoxy matrix).

Recent synchrotron experiments have demonstrated, for the first time, the feasibility of mapping lattice strain inside CFRP laminates using high‐energy WAXS.^[^ ^33^, ^36^ ^]^ In one such study, researchers scanned a unidirectional CFRP specimen containing a curved “hump‐back” defect and successfully measured the 002 and 100 reflection shifts of carbon fibers, identifying localized residual‐strain concentrations in the wrinkle region. These pioneering works illustrate that WAXS can non‐destructively sense internal stress perturbations caused by fibers misalignment or thermal‐cure effects. However, WAXS alone cannot visualise the actual cracks or delamination associated with a wrinkle, and thus correlating those strain hotspots to physical damage remains a challenge.^[^ ^14^ ^]^ To date, very few studies have combined 3D imaging and diffraction on the same composite specimen, particularly for polymer composites. Therefore the link between a wrinkle's geometry, the induced stress state, and the sequence of damage mechanisms has not yet been fully resolved in the literature.^[^ ^14^, ^33^, ^34^ ^]^

Despite recent demonstrations that synchrotron X‐ray diffraction can map lattice strain in CFRP, including load‐dependent mapping of the {002} and {100} reflections, most studies stop at feasibility and orientation metrics rather than scalable, point‐wise microstrain quantification across multiple load steps.^[^ ^33^, ^37^ ^]^ Automated, end‐to‐end peak extraction over large spectral datasets with rigorous per‐point $d_{0}$ referencing is seldom documented.^[^ ^38^, ^39^ ^]^ Joint acquisitions co‐registering WAXS with XCT on the same specimen are emerging but remain limited in scope and, to date, rarely couple microstrain mapping with orientation‐aware predictive models of wrinkle‐induced strain redistribution;^[^ ^40^ ^]^ related WAXS/SAXS studies typically prioritise orientation over microstrain.^[^ ^41^, ^42^ ^]^

This study advances a co‐registered synchrotron XCT–WAXS investigation on hybrid woven carbon–glass/epoxy coupons with controlled wrinkles, executed in a single experimental campaign at Diamond Light Source on Beamline B16. The B16 configuration enables rapid mode‐switching between full‐field tomography and rastered diffraction without repositioning the sample, preserving a strict one‐to‐one correspondence between tomographic features and diffraction measurements.^[^ ^33^, ^34^ ^]^ This pairing yields complementary datasets: XCT captures microstructure and damage in real space, while WAXS provides reciprocal‐space crystallography and lattice strain from the carbon fibers. The dual‐technique design exposes behaviors inaccessible to a single modality: XCT localises matrix cracking, delamination and fibers kinking, while co‐registered WAXS indicates whether those regions exhibited abnormal fiber strains or misorientation beforehand, linking wrinkle geometry to stress redistribution and early‐stage damage precursors.

Three specimen classes (pristine, small‐wrinkle, large‐wrinkle) were manufactured and tested under in situ quasi‐static compression while alternating XCT and WAXS without disturbing registration. The turbostratic graphitic structure motivates the two reflections central here: $d_{002}$ senses inter‐layer spacing (radial to the fibers axis) and $d_{100}$ probes basal‐plane spacing (axial), reported as $\varepsilon_{002}$ and $\varepsilon_{100}$, respectively.

### 1.1 Industrial Relevance

Combined XCT‐WAXS, as implemented in this study, offers unprecedented resolution for investigating the influence of defects on the deformation and failure of carbon fibers composite parts. This technique enables unique insights into microscale material behavior, which in turn supports the development of quantitative analytical models capable of reliably predicting material response under load for specific part geometries.

Non‐destructive testing (NDT) of structural aerospace components is now routinely used to identify defects exceeding defined size and shape thresholds.^[^ ^10^, ^14^, ^15^ ^]^ The enhanced insight provided by XCT‐assisted WAXS allows, for the first time, reliable prediction of localized knock‐down effects in both stiffness and strength.^[^ ^14^ ^]^ These quantitative descriptors can then be integrated into existing macroscale simulations, enabling engineers to more accurately assess the impact of defects on part performance.

This approach facilitates a more scientifically informed decision‐making process regarding part acceptance, helping to refine rejection criteria and reduce both financial and environmental costs associated with composite part production.

### 1.2 Paper Road‐Map

Section 2 outlines the experimental design and end‐to‐end analysis pipeline, from materials and in‐situ compression (Section 2.1), through co‐registered XCT–WAXS acquisition and reduction, to lattice‐strain mapping and the orientation‐aware predictive analytical model (Sections 2.7, 2.8). Section 3 presents the main results and discussion, field maps of lattice spacing and strain, load‐dependent trends, wrinkle‐induced localisation, and a model–measurement comparison (Section 3). Section 4 concludes with key findings, practical implications for inspection/design, and an outlook.

## 2 Experimental Section

### 2.1 Materials, Specimen Preparation, and In Situ Compression Testing

Hybrid woven carbon–glass/epoxy laminates with a nominal cured thickness of 2.0mm were supplied by Dowty Propellers (Gloucester, UK). The reinforcement comprised a woven hybrid fabric combining carbon and glass tows. Unless otherwise stated, the warp (loading) direction defines the global x‐axis, with y the in‐plane transverse (weft) and z the through‐thickness direction. As with any laminated composite, small manufacturing imperfections (e.g., micro‐voids or slight tow waviness) may be present, however no gross porosity, dry patches, or foreign inclusions were evident on visual inspection of the seed 50mm × 50mm panels.

**Figure 1:**
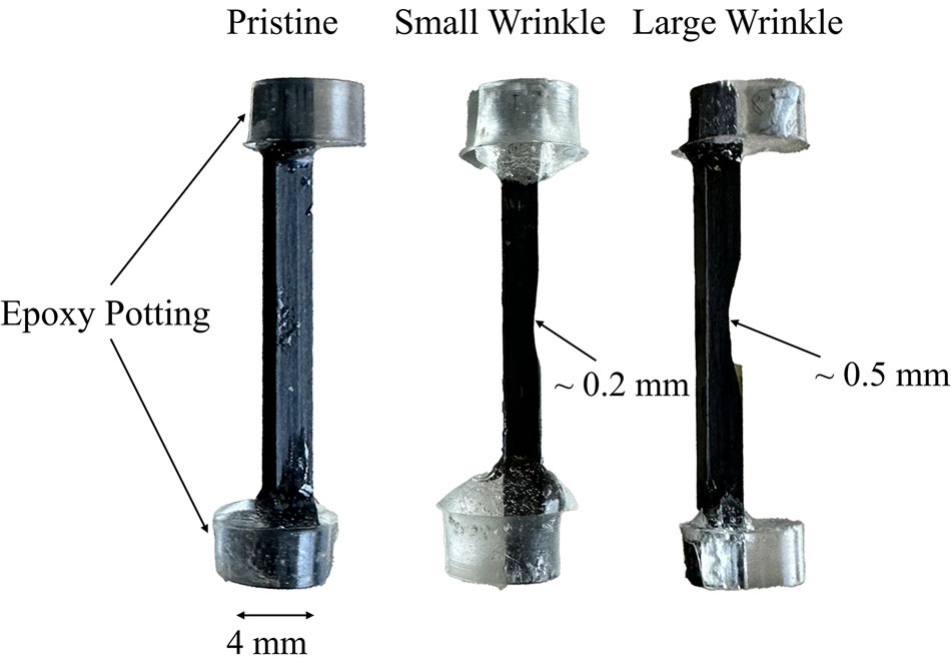
Visual comparison of the three specimen variants used in this study: pristine (left), small‐wrinkle (centre) and large‐wrinkle (right). All specimens are epoxy‐potted at both ends; the out‐of‐plane wrinkle amplitude increases from negligible to ≈ 0.2 and 0.5mm, respectively. Scale bar: 2mm.

Three specimen variants were manufactured (**Figure** **1**): pristine (no intentional defect), small‐wrinkle, and large‐wrinkle. For the wrinkled cases, a local out‐of‐plane undulation was introduced in the outer woven ply (warp aligned with x) by placing a shaped compliant shim beneath the ply prior to consolidation. The target peak amplitude at the outer ply was ≈ 0.2mm for small‐wrinkle and ≈ 0.5mm for large‐wrinkle. The wrinkle centroid was positioned nominally at mid‐gauge as shown inFigure 1.

From each 50mm × 50mm panel, 25mm × 25mm blanks were cut by wire‐sawing along and transverse to the warp direction in order to minimise tow damage. Coupons were then finish‐machined to a gauge length of 25mm with a square cross‐section of 2mm × 2mm (loading axis ∥ warp tows). Edges were de‐burred and progressively polished with SiC abrasive papers to remove machining notches and to minimise edge‐induced stress concentrations.

**Figure 2:**
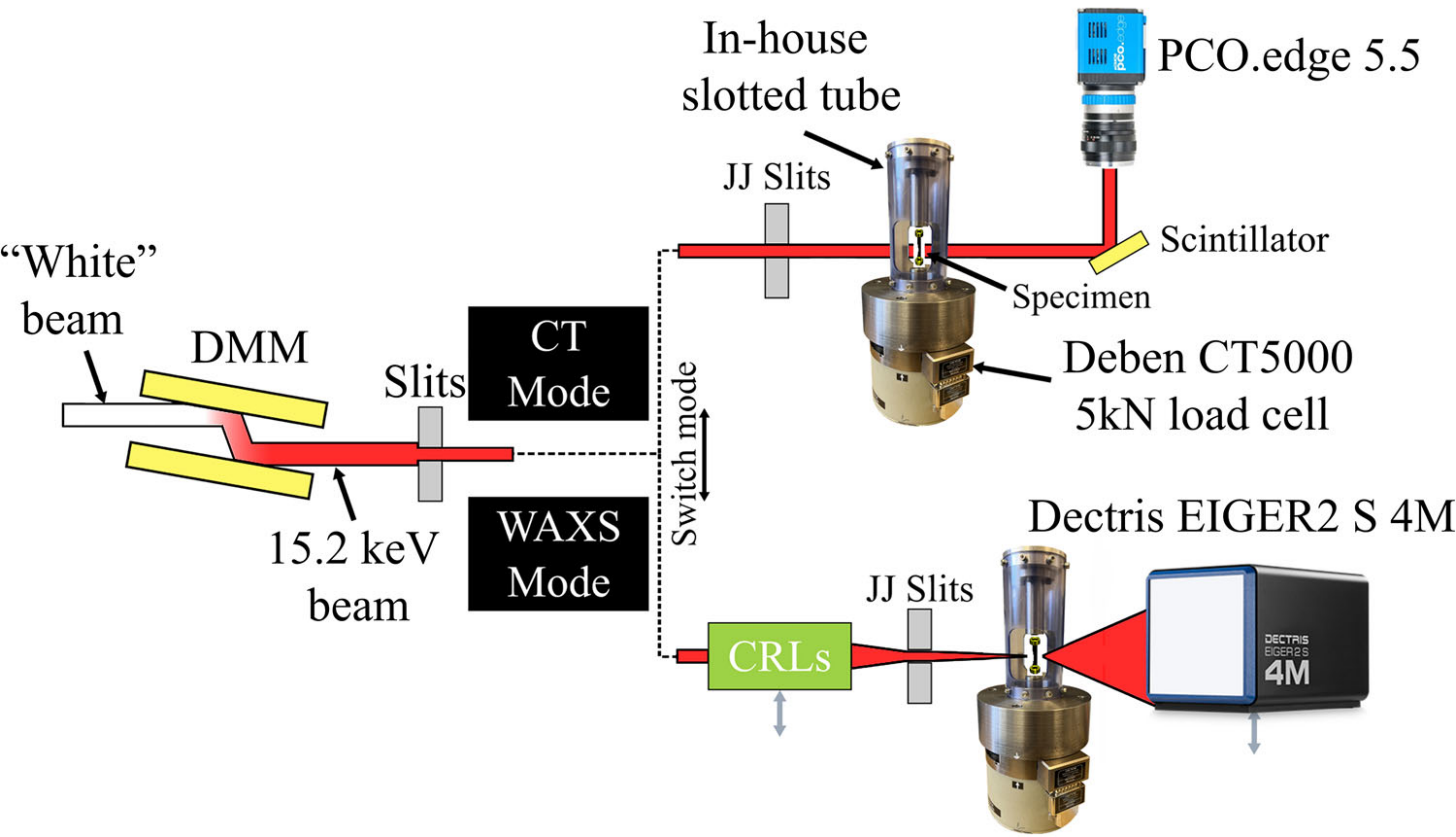
Dual‐mode CT/WAXS setup on beamline B16: DMM beam at 15.2 keV, CT mode is shown via the top path, WAXS mode was achieved via CRL focusing onto the sample and the use of the Dectris EIGER2 S 4M. The Deben CT5000 stage is used for in situ loading, and is rastered in front of the stationary beam.

To ensure coaxial loading and reproducible clamping in the in‐situ compression rig (**Figure** **2**), coupon ends were potted in a 3D‐printed alignment jig using EL2 epoxy with AT30 hardener (Easy Composites, UK) following the manufacturer's instructions. The jig provided self‐centering guides, datum flats for parallelism, and bleed channels while maintaining a low X‐ray attenuation path around the gauge section. A short potted length was used at each end, leaving an unpotted gauge of 25mm. During potting, the warp‐parallel reference face was held against a datum to align the x‐axis with the loading axis, and end‐face squareness was checked before cure. The potting resin cured for 24h at ambient conditions (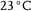 ± 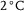, 50/cent ± 5/cent RH), followed by a 6h post‐cure at 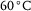.

After potting, the gauge surfaces were lightly re‐polished and examined under a stereo microscope to verify edge quality. Coupons were pre‐screened with a low‐resolution laboratory XCT scan to confirm wrinkle placement and to exclude unintended defects. Specimens exhibiting delaminations or foreign‐object inclusions within the gauge section were rejected.

Prior to the in situ campaign on beamline B16, a series of destructive compression tests were conducted on coupons from each class to establish representative failure loads and damage mechanisms. Based on these pre‐tests, load levels for the in situ XCT/WAXS experiments were selected to ensure observable damage evolution without immediate catastrophic failure. Three representative previously unloaded test specimens, one pristine, one small‐wrinkle, and one large‐wrinkle–were subsequently tested in situ.

The 2mm × 2mm cross‐section and 25mm gauge length were chosen to i) ensure the results were not dominated by edge effects, ii) ensure adequate X‐ray transmission and signal‐to‐noise ratios at the beam energies used, iii) suppress global Euler buckling in compression while permitting local kinking/tow microbuckling where applicable, and iv) match the field of view for rapid, full‐field XCT on beamline B16 without repositioning between imaging and diffraction modes.

In situ compression tests were performed on beamline B16 (Diamond Light Source) using a Deben CT5000 micro‐compression stage (5kN). Coupons were aligned coaxially with the tomography rotation axis and loaded under quasi‐static displacement control at 0.1mmmin ^−1^ (Figure 2). During each in situ acquisition, the load cell position was held constant. This displacement‐controlled approach ensured specimen stability throughout CT and WAXS scans, preventing motion artefacts and preserving spatial registration. Load–hold levels were specimen‐class specific (**Table** **1**). Failure was flagged by the CT5000 controller via automatic load‐drop/ overload detection (Figure 2). In CT mode, the PCO.edge 5.5 camera is positioned vertically above the sample and images an angled scintillator screen, which converts X‐rays into visible light. This setup avoids placing the camera directly in the beam, allowing higher flux on the sample while protecting the detector. It also enables flexible magnification via vertically translatable optics, and facilitates compact integration with other detectors such as the EIGER2 for dual‐mode analysis.

**Table 1:** Load–hold schedule by specimen class. Column headers list load (N, top) and nominal compressive stress (MPa, bottom) for a 2mm × 2mm gauge cross‐section. A ✓ denotes a scheduled hold/measurement.

**Specimen**	0N (0MPa)	200N (50MPa)	400N (100MPa)	600N (150MPa)	800N (200MPa)	1100N (275MPa)	1200N (300MPa)
Large wrinkle	✓	✓	✓	✓	✓	✓	
Small wrinkle	✓		✓		✓		✓
Pristine	✓		✓		✓		✓

### 2.2 XCT–WAXS Acquisition, Calibration, and Reconstruction

Full‐field CT used a 15.2keV beam selected by a double‐multilayer mirror (DMM). Upstream JJ slits defined the illuminated footprint and reduced parasitic scatter onto the detector. Projections were recorded on a PCO.edge 5.5 with 4× optical magnification, giving an isotropic voxel size of 1.5μm.

The rotation stage used in the experiment has a precision of $0.01^{\circ }$, and the scan was performed using this increment to collect 18 000 projections over $180^{\circ }$. This was determined through setup tests that balanced angular step size and exposure time to optimise reconstruction quality. The final settings of $0.01^{\circ }$ steps and 0.06s exposure produced high‐quality tomographs within an acceptable acquisition time of 18 min. This corresponds to a cumulative detector exposure of 1080s. The total scan duration, including stage rotation and detector readout overheads, was 20 to 25 min. At each load step, dark and flat frames were acquired prior to CT to reducing shading and ring artefacts; a dedicated ring‐artefact suppression filter was then applied during reconstruction.

Immediately after each CT scan, the Compound Refractive Lenses (CRLs) and diffraction detector (an EIGER 2 S 4M) were translated into the beam. The same 15.2keV beam was directed into the CRLs to achieve a nominal circular spot of diameter 10μm. The downstream JJ slits remained in place to reduce parasitic scatter. Wide‐angle X‐ray Scattering (WAXS) patterns were collected on an EIGER 2 S 4M. After LaB_6_ calibration, the sample–detector distance (SDD) was determined to be 117.19mm and was used for all subsequent data reductions.

In order to collect WAXS data over the wrinkle‐affected regions identified by XCT, 2D raster scans were executed. The specimen was rastered at 150μm in the vertical and horizontal directions, which set the resolution of the resulting strain field maps. At each load a sample grid of 67×(17) (1139 positions) was collected. The exception to this was the large‐wrinkle specimen at 1100N for which an extended map of 67×31 (2077 positions) was used to capture the larger size of the specimen that was induced after failure. The exposure time was selected to achieve a minimum on‐peak signal‐to‐noise ratio for both {002} and {100} reflections; this was found to be 8s.

Calibration of the diffraction geometry was performed in DAWN,^[^ ^38^ ^]^ using lanthanum hexaboride LaB_6_ powder (SRM 660c from the U.S. National Institute of Standards and Technology).^[^ ^43^, ^44^ ^]^ A static mask excluded the beamstop shadow, module gaps, dead/hot pixels and parallax‐affected edges. Debye–Scherrer rings were auto‐located and tidied before non‐linear least‐squares refinement of beam centre $\left(x_{0},y_{0}\right)$, beam energy, SDD and small detector tilts (pitch, roll). Iterative outlier rejection proceeded until the radial RMS misfit fell below one pixel and azimuthal residuals showed no systematic trend. The refined detector geometry and static mask were batch‐applied to all frames to convert pixel coordinates to diffraction space.

At each prescribed load hold the sequence *CT data collection* → *insert focusing optics and diffraction detector* → *WAXS raster* → *remove focusing optics and diffraction detector* was implemented. Low‐attenuation fiducials placed during specimen preparation enabled alignment of maps with the 3D CT volume without intercepting the diffracted beam, ensuring that microstructural features segmented in CT (e.g. local fibers‐path undulation) could be analysed against co‐located lattice‐spacing/orientation fields from WAXS.

Reconstructions were performed in Savu with dark/flat correction, ring‐artifact suppression, and GPU‐accelerated filtered back‐projection (Ram–Lak filter).^[^ ^45^, ^46^ ^]^ For low‐contrast plies, single‐distance Paganin phase retrieval (δ/β=1500 at E=15.2keV) was applied only where explicitly stated; otherwise, conventional absorption reconstructions were used.^[^ ^32^, ^47^ ^]^ The reconstructed volumes were examined slice‐by‐slice in ImageJ/Fiji to localise failure features (e.g. kink bands, delamination initiation/propagation) and to select representative sections for reporting.

### 2.3 XCT‐Assisted WAXS and Data Alignment

An alignment procedure was used to correlate XCT and WAXS on a point‐by‐point basis. The in situ dual‐mode configuration on B16 kept the specimen fixed on the same stage while alternating between tomography and diffraction (Figure 2), avoiding any repositioning. This preserved a one‐to‐one spatial correspondence between tomographic features and diffraction sampling, enabling direct comparison of CT‐identified structure (e.g. wrinkle geometry, damage) with co‐located WAXS‐derived fields (lattice spacing, strain, orientation).

#### 2.3.1 Fiducial‐Based Coordinate Calibration

Low‐attenuation fiducial markers were embedded to provide common references visible in the CT volume. Their 3D positions in the reconstruction defined anchor points that were matched to the WAXS raster (stage motor coordinates). A rigid transformation was applied to align XCT voxels to the WAXS grid by matching fiducial locations. The specimen orientation was unchanged during mode switching, which meant that the transform was reduced to an in‐plane translation with negligible rotation or scale. Fiducials were positioned outside the region of interest and clear of the diffracted beam path. This step anchored both modalities to a shared (x,y) frame so each diffraction point could be located in the CT stack.

#### 2.3.2 Drift Correction by Global Translation

Small inter‐scan drifts (e.g. mechanical creep under load) were corrected at each load step by estimating a single in‐plane shift (Δx,Δy) between the loaded and baseline (0N) WAXS rasters. The shift was obtained as the median nearest‐neighbor offset between raster coordinates (within a radius tied to the baseline point spacing). Only a pure translation was applied, preserving the WAXS grid geometry. If reliable matches were insufficient, the conservative (Δx,Δy)=(0,0) was adopted and the step flagged for quality control (QC). This automatic correction maintained registration to the 0N reference without manual tweaking.

#### 2.3.3 Interpolation of the Zero‐Load Reference

Lattice strain at load requires comparison to the local zero‐load spacing $d_{hkl}^{0}\left(x,y\right)$. Loaded and baseline rasters are not necessarily identical, therefore $d_{hkl}^{0}$ was interpolated at each aligned loaded coordinate using inverse‐distance weighting (IDW). Up to k nearest baseline neighbors within a search radius R were used, with weights $w_{i}\propto r_{i}^{-p}$ (distance to the ith neighbor $=r_{i}$). The IDW hyperparameters (p,k,R) and the drift‐registration radius were chosen per specimen based on the baseline sampling density (see Table 3). Per‐point engineering lattice strain was then formed as in Equation (19).

**Table 2:** Fixed configuration for extracting d‐spacings from the caked maps I(χ,q). Angles follow the DAWN convention of $\left[0,360^{\circ }\right]$.

Reflection	Sector centres	Half‐width Δ	q‐window	Spike filter
{002}	$180^{\circ },\;360^{\circ }$	$12.5^{\circ }$–$30^{\circ }$	$\left[1.55,\;1.97\right]\;{Å}^{-1}$	MAD threshold k≈12
{100}	$90^{\circ },\;270^{\circ }$	$\approx 90^{\circ }$	$\left[2.80,\;3.30\right]\;{Å}^{-1}$	MAD threshold k≈12

**Table 3:** Registration / IDW hyperparameters used for each specimen where h is the median nearest–neighbor spacing of the baseline raster (effective grid step), $r_{\mathrm{NN}}=\alpha h$ is used for registration and R=βh for IDW neighborhoods.

Specimen	h [mm]	α	p	k	β
Large‐wrinkle	0.15	1.00	2.0	16	2.0
Small‐wrinkle	0.14	1.10	1.9	18	2.1
Pristine	0.16	0.90	2.1	14	1.9

#### 2.3.4 Accuracy and Preservation of Spatial Correspondence

The workflow relies only on rigid alignment (fiducials and global translation) and neighbor‐based interpolation of the baseline field; no warping or re‐meshing of the WAXS data was performed. Consequently, geometric relationships observed in CT (distances, angles, wrinkle footprint) are preserved when overlaid with WAXS maps, and spatial fidelity is limited primarily by the measurement resolutions (≈10μm WAXS spot; CT voxel size). This ensured that microstrain hot‐spots identified in WAXS could be overlaid directly onto CT slices/volumes at the same physical locations, enabling the correlated interpretation reported in Section 3.5.

### 2.4 WAXS Reduction, Peak Extraction, and Frame Classification

**Figure 3:**
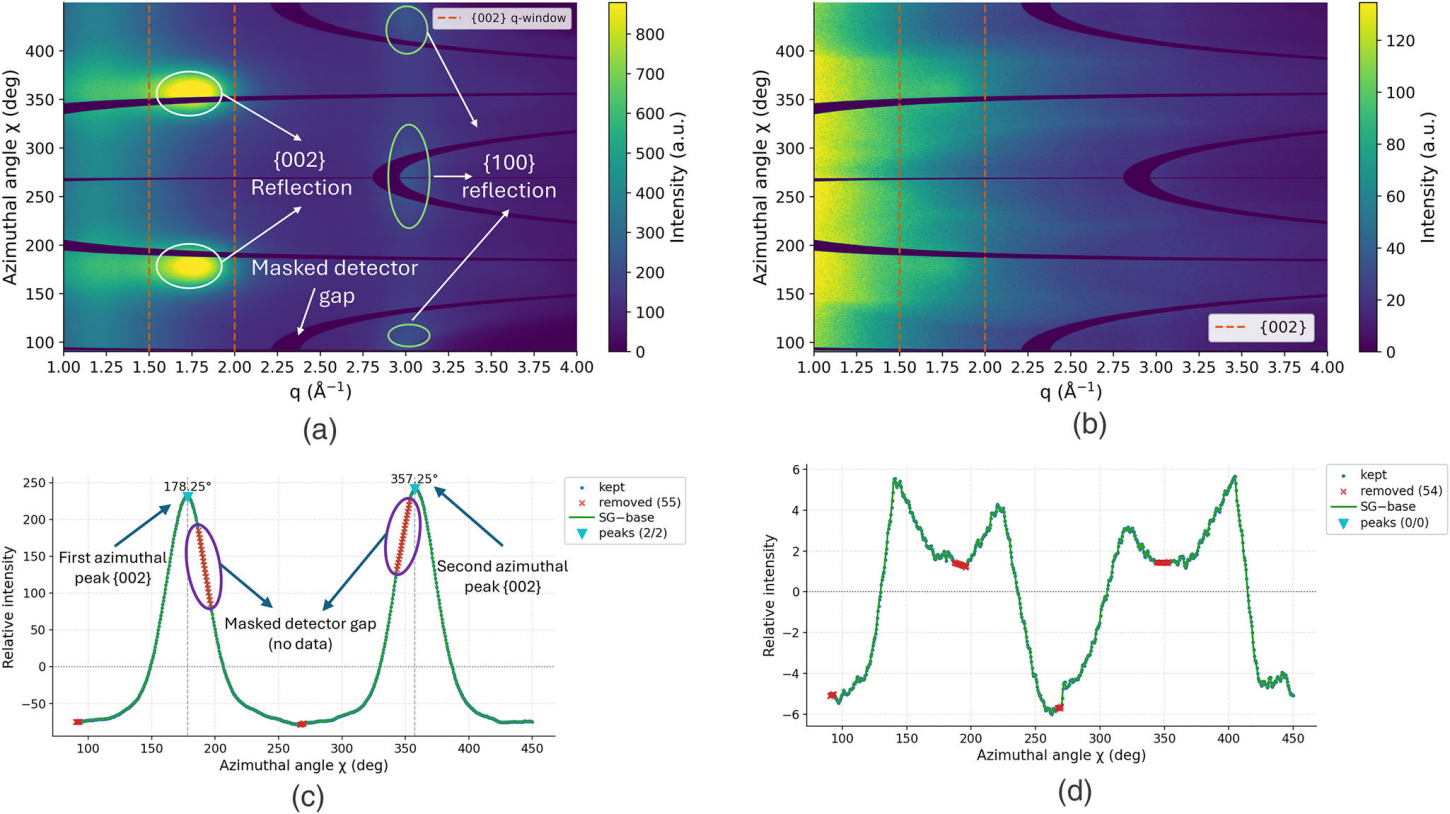
Caked maps and azimuthal‐profile for fibers vs resin frames. a) Caked intensity I(χ,q) for a fibers frame showing {002} lobes (white ellipses) and weaker {100} signal; orange dashed lines mark the fixed {002} q‐window. b) Azimuthal profile from (a) after de‐spiking and Savitzky–Golay smoothing; a first‐order Fourier background B(χ) is subtracted to yield flattened signal $F\left(\chi \right)=I_{\mathrm{SG}}\left(\chi \right)-B\left(\chi \right)$. c) Caked I(χ,q) for a resin/air frame using the same {002} window shows no lobes. d) Flattened profile from (c) remains peak‐free; frame is labeled resin/air.

The resulting WAXS images were caked in dawn/pyFAI to produce intensity maps I(χ,q) with appropriate masking, polarisation, and solid‐angle corrections.^[^ ^48^ ^]^ The caked data have the form $N_{\chi }\times N_{q}$ with axes χ (azimuthal angle, degrees) and q, where q is the scattering vector defined by $q=\frac{2\pi }{d}$. **Figure** **3** illustrates the link between the caked map and the corresponding azimuthal profile, as well as the profile processing.

As noted in prior studies,^[^ ^33^ ^]^ the nature of the modern photon counting detectors means that there are typically gaps within the diffraction profile which are masked from the data set. This results in a “missed” region within the diffraction pattern, as can be seen in Figure 3a,c. For crystalline materials the sharp nature of the peak either means that a specific peak can be missed or curtailed, and therefore these regions are typically not included in the assessment of the pattern. However, in semi‐crystalline materials of the type presented in this study, it is almost guaranteed that part of the broad peak of interest falls within one or more of these regions. Therefore, a more careful and complex approach is required to interpret these peaks effectively. A refined peak identification and fitting pipeline was therefore developed as outlined below.

For each frame, an azimuthal profile is formed by integrating the caked intensity over a fixed q window that isolates the {002} scattering; the default is $\left[q_{\min},q_{\max}\right]=\left[1.50,2.00\right]\;{Å}^{-1}$. This window is identical for all frames, and therefore absolute intensities are left un‐normalized.

(1) $$I\left(\chi \right)\;\approx \;\int_{q_{\min}}^{q_{\max}}I\left(\chi ,q\right)\;dq$$

Spurious samples are first limited by a floor $I\left(\chi \right)\geq I_{\min}$. Central‐difference derivatives with circular wrap are then formed using the median azimuthal step Δχ:

(2) $$I^{\prime }\left(\chi_{i}\right)\;\approx \;\frac{I\left(\chi_{i+1}\right)-I\left(\chi_{i-1}\right)}{2\Delta \chi },\;I^{\prime \prime }\left(\chi_{i}\right)\;\approx \;\frac{I\left(\chi_{i+1}\right)-2I\left(\chi_{i}\right)+I\left(\chi_{i-1}\right)}{\Delta \chi^{2}}$$

Robust scales are computed by the median absolute deviation (MAD),^[^ ^49^ ^]^

(3) $$\mathrm{MAD}\left(x\right)\;=\;1.4826\;\mathrm{median}\;\left(\;|x-\mathrm{median}(x)|\;\right),$$

and converted to z–scores,

(4) $$z_{1}=\frac{|I^{\prime }\left(\chi \right)-\mathrm{median}\left(I^{\prime }\right)|}{\mathrm{MAD}(I^{\prime })},\;z_{2}=\frac{|I^{\prime \prime }\left(\chi \right)-\mathrm{median}\left(I^{\prime \prime }\right)|}{\mathrm{MAD}(I^{\prime \prime })}$$

Samples with $z_{1}>k$ (optionally also $z_{2}>k$) are rejected; here k=8.50.

Rejected points are circularly infilled; the profile is then smoothed on the circle by a Savitzky–Golay filter.^[^ ^50^ ^]^ A first‐order Fourier fitted background with fixed phase origin $\chi_{0}$ is fitted and subtracted:

(5) $$B\left(\chi \right)=a_{0}+a_{1}\cos\theta +b_{1}\sin\theta ,\;\theta =\frac{2\pi }{360^{\circ }}\left(\chi -\chi_{0}\right)$$

yielding the flattened signal

(6) $$F\left(\chi \right)=I_{\mathrm{SG}}\left(\chi \right)-B\left(\chi \right)$$

Candidates are local maxima of F(χ). Acceptance requires all of the following:

(7) $$\begin{array}{cccc} & \text{(prominence)} & & \;\;\;\;F\left(\chi_{i}\right)\;\geq \;\kappa \;\mathrm{MAD}\;\left(F(\chi )\right),\;\kappa =2.0,\\ & \text{(minimum width)} & & \;\;\mathrm{FWHM}\left(\chi_{i}\right)\;\geq \;21^{\circ },\\ & \text{(minimum separation)} & & \;\;\;\;|\chi_{i}-\chi_{j}|\;\geq \;160^{\circ }\;\;\text{for all}\;i\neq j\;\text{(on the circle)},\\ & \text{(raw intensity check)} & & \;\;\;\;I\left(\chi_{i}\right)\;\geq \;50\left(a.u\right)\;\text{(guards against flattening artefacts)}. \end{array}$$

Let $N_{p}$ be the number of accepted peaks in the {002} azimuthal profile. The frame label is

(8) $$\mathrm{label}\;=\;\left\{\begin{array}{cc} \mathrm{fiber}, & N_{p}=2,\\ \mathrm{resin}/\mathrm{air}, & N_{p}\neq 2\; \end{array}\right.$$

The two accepted peaks represent the two lobes of the {002} fibers reflection and are expected to lie roughly opposite each other; a separation threshold of $160^{\circ }$ tolerates modest misalignment while rejecting spurious multi‐peak textures. A minimum width of $21^{\circ }$ guards against narrow, non‐physical spikes. Figure 3 links the caked I(χ,q) map to I(χ); Figure 3 illustrates the filtering, flattening, and classification stages on representative fibers and resin/air frames.

### 2.5 Fiber Orientation

Fiber orientation extracted from the two‐lobed {002} azimuthal texture provides a signed misalignment field Δ that underpins three later steps: i) it is consistent with the fibers/resin frame classification of Section 2.4 (two‐lobe texture), ii) it contextualises d‐spacing and lattice‐strain localisation in Sections 2.6, 2.7, and iii) it supplies the orientation channel γ for the predictive model in Section 2.8. In turbostratic graphitic fibers, the {002} planes (normal to the fibers axis) concentrate scattering into two equatorial lobes roughly $180^{\circ }$ apart on the caked ring. The lobe centroids align with the local fibers axis; hence, a local misalignment rotates both lobes together. Peak width primarily reflects orientation dispersion (e.g. tow waviness); centroid location sets the local axis used downstream.

Local fiber orientation was identified from the two‐lobed azimuthal texture of the graphitic {002} scattering. For each raster position (“frame”) classified as fibers in Section 2.4, the azimuthal profile I(χ) was obtained from the caked map I(χ,q) by integrating over $\left[q_{\min},q_{\max}\right]=\left[1.50,\;2.00\right]\;{Å}^{-1}$ as in Equation (1).

The measured intensity was described by a low‐order Fourier background combined with either an axial twofold von Mises texture or a two‐peak form. With $\phi =\chi \;\mathrm{mod}\;360^{\circ }$, the background model used was

(9) $$B\left(\phi \right)=\beta_{0}+\sum_{k=1}^{K}\left[\beta_{2k-1}\cos\left(k\phi \right)+\beta_{2k}\sin\left(k\phi \right)\right],\;K\leq 2$$

The axial representation used

(10) $$I_{\mathrm{axial}}\left(\phi \right)=B\left(\phi \right)+C\exp\;\left[\kappa \cos2(\phi -\mu )\right]$$

where μ is the fibers‐axis direction (defined modulo $180^{\circ }$), C>0 is a scale factor related to peak intensity, and κ>0 is a concentration parameter. The two‐peak representation used

(11) $$I_{2\mathrm{pk}}\left(\phi \right)=B\left(\phi \right)+A_{1}\exp\;\left[\kappa \cos(\phi -\mu )\right]+A_{2}\exp\;\left[\kappa \cos\left((\phi -\mu )-\pi \right)\right]$$

with lobe amplitudes $A_{1},A_{2}>0$ and a shared concentration κ. Initial values were seeded from the classified peak pair $\left(p_{1},p_{2}\right)$ using $\mu_{0}=∠\;\left(e^{ip_{1}}+e^{i(p_{2}-\pi )}\right)$ and a $\kappa_{0}$ corresponding to a nominal $\mathrm{FWHM}\approx 20^{\circ }$. Parameters were obtained by bounded non‐linear least squares (SciPy least_squares) with linear loss, $\kappa \in [10^{-3},10^{3}]$, and K=2. An amplitude‐independent angular width was also reported via the equivalent von Mises full width at half maximum,

(12) $${\mathrm{FWHM}}_{\mathrm{eq}}=2\arccos\;\left(1-\frac{\ln2}{\kappa }\right)$$

which was applied uniformly to Equations (10) and (11).

**Figure 4:**
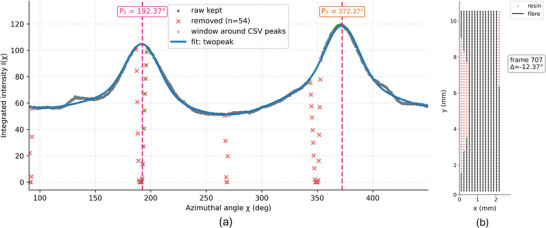
Fiber orientation from the two‐lobe {002} texture. a) Azimuthal profile I(χ) integrated over $q=\left[1.50,2.00\right]\;{Å}^{-1}$; a two‐peak von Mises fit with centroids $p_{1},p_{2}$ yields the signed misalignment Δ. b) Stick map of Δ across the raster: black sticks show the local fiber axis $\theta =90^{\circ }+\Delta$ (positive = counter‐clockwise); red dots mark resin/air frames excluded from fiber analysis.

The angular offset Δ of the peak was subsequently defined relative to the adopted geometry in which an ideal vertical fibers produces {002} lobe centroids at $\left\{180^{\circ },360^{\circ }\right\}$ (symmetry axis $90^{\circ }$). A representative single‐frame azimuthal fit alongside the spatial stick map of Δ is shown in **Figure** **4**.

### 2.6 Quantification of d‐Spacing

The graphitic {100} (axial) and {002} (radial) d‐spacings were obtained directly from the caked intensity I(χ,q) produced by the DAWN/pyFAI pipeline in Section 2.4. Here, q is the magnitude of the scattering vector, computed from the refined geometry via

(13) $$\begin{array}{cc} & q\;=\;\frac{4\pi }{\lambda }\;\sin\theta ,\\ & \;\text{with}\;\lambda \;\text{the calibrated X-ray wavelength and}\;\theta \;\text{the Bragg angle,} \end{array}$$

and peak centres $q_{0,hkl}$ were converted to lattice spacings by

(14) $$d_{hkl}\;=\;\frac{2\pi }{q_{0,hkl}}$$

Only raster positions classified as fibers were processed so that peak finding and fitting operated exclusively on fibers‐bearing frames.

**Figure 5:**
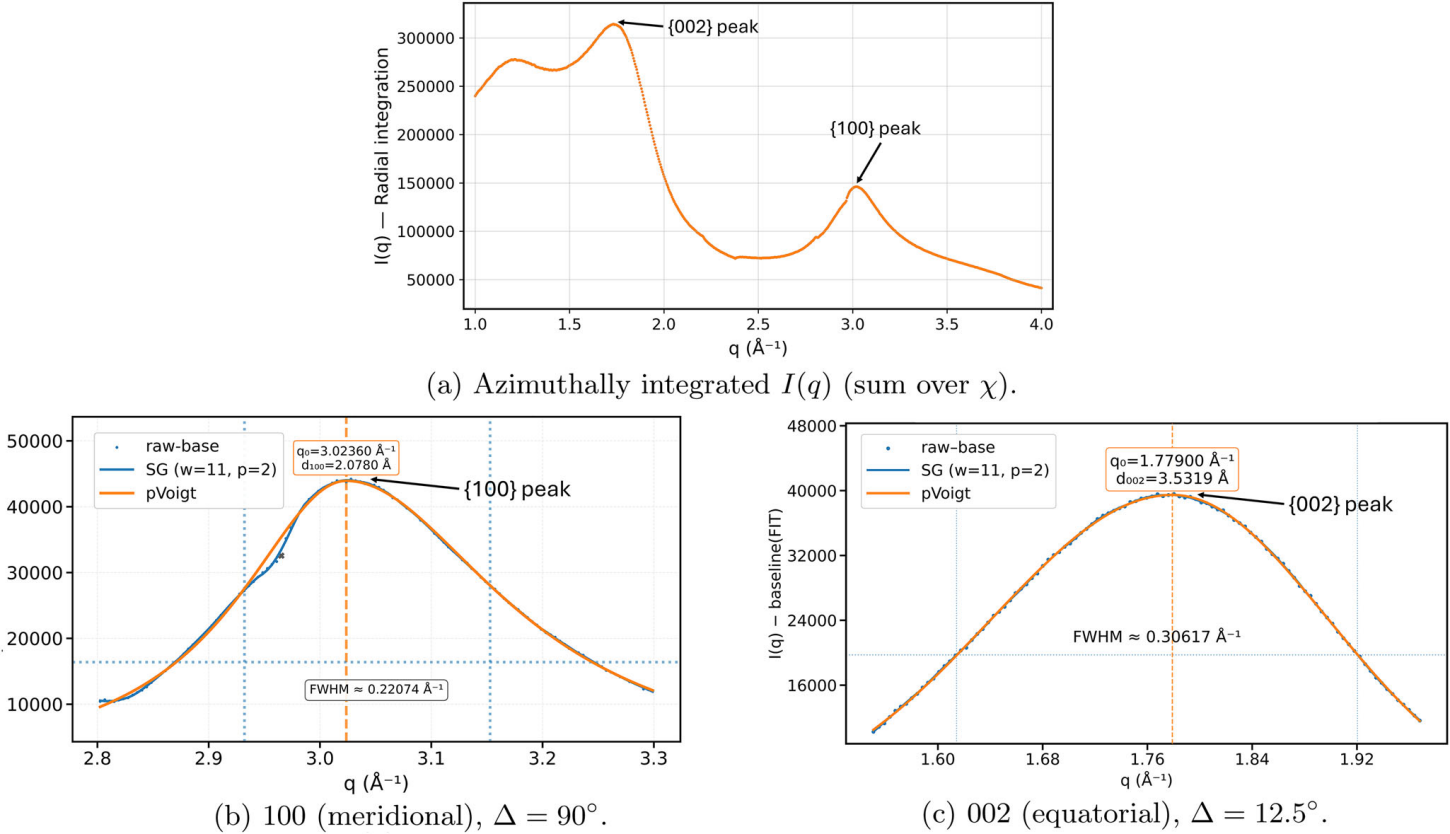
Peak selection and split pseudo‐Voigt fitting on a representative fibers frame: a) azimuthally integrated profile I(q) highlighting the dominant 002 and 100 peaks; b) 100 meridional; c) 002 equatorial. Background‐subtracted intensities (blue: linear fitted background a+bq with Savitzky–Golay smoothing for seeding; orange: final split pseudo‐Voigt fits). Dashed vertical lines indicate fixed q‐windows; dotted lines mark FWHM.

**Figure 6:**
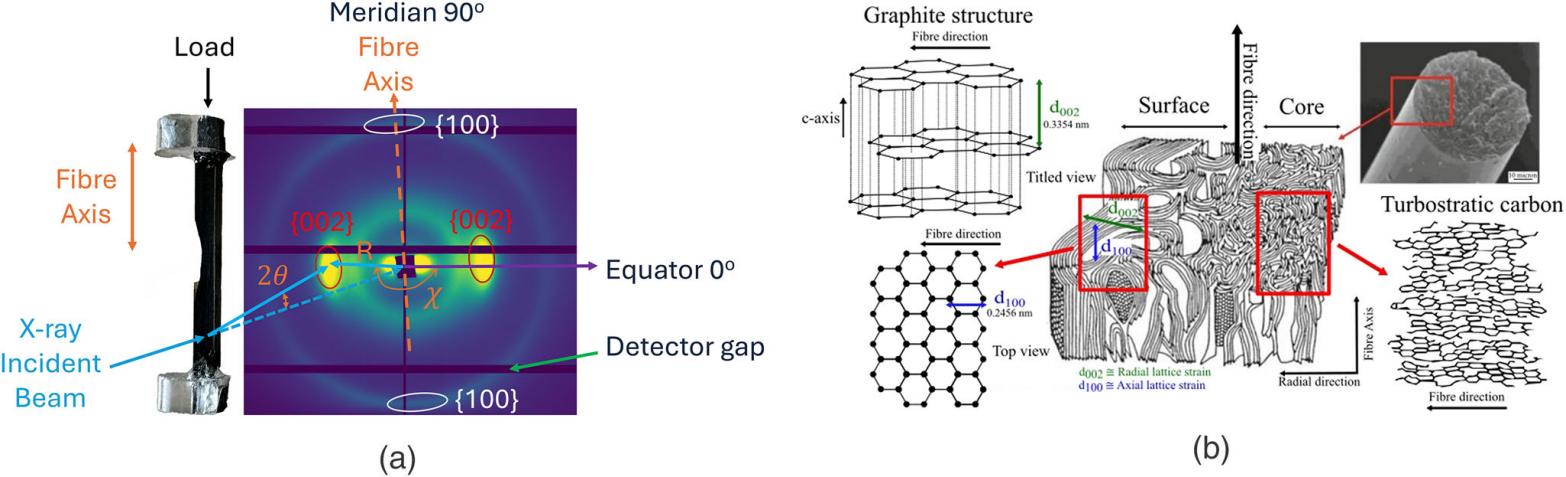
Sample diffraction pattern and fibers structure a) Sample orientation and corresponding diffraction pattern with fibers axis and resulting {002} / {100} peaks highlighted. b) Schematic of carbon fibers atomic structure linking reciprocal‐space features to real‐space lattice spacings: $d_{002}$ (interlayer, radial to the fiber axis) and $d_{100}$ (in‐plane, axial to the fiber axis), adapted from.^[^ ^33^ ^]^

**Figure** **5** shows, on a representative fibers frame, the peak extraction process which includes background removal, Savitzky–Golay smoothing, and split pseudo‐Voigt fitting of the {100} (meridional, $\Delta =90^{\circ }$) and {002} (equatorial, $\Delta =12.5^{\circ }$) reflections. The relative positions of these peaks on the 2D WAXS pattern are shown in **Figure** **6**. This is coupled with the experimental geometry and the physical link to the carbon‐fibers microstructure: the equatorial {002} lobes track the fibers axis and provide the *radial* inter‐layer spacing $d_{002}$, while the meridional {100} response provides the *axial* basal‐plane spacing $d_{100}$ in turbostratic graphite.^[^ ^33^ ^]^

For each fibers frame, sector‐integrated radial profiles were formed by summing intensities over azimuthal windows that isolate the fibers texture of the target reflection. For {002}, two narrow sectors centred at $\chi \approx \{180^{\circ },\;360^{\circ }\}$ (DAWN convention) with half‐width $\Delta \in [12.5^{\circ },\;30^{\circ }]$ captured the two fibers lobes while suppressing background. For {100}, meridional sectors centred at $\chi \approx \{90^{\circ },\;270^{\circ }\}$ with a larger half‐width ($\Delta \approx 90^{\circ }$) improved signal‐to‐noise for this more diffuse and lower‐intensity peak. The scattering angles were converted to q using Equation (13) (here λ=0.815689 Å). The resulting sector‐summed profile is

(15) $$I_{\mathrm{rad}}\left(q\right)\;=\;\sum_{\chi \in S}I\left(\chi ,q\right)$$

where S denotes the union of the two azimuthal sectors for the reflection under consideration. Fixed q‐windows were used across frames: $\left[1.55,\;1.97\right]\;{Å}^{-1}$ for {002} and $\left[2.80,\;3.30\right]\;{Å}^{-1}$ for {100}. This peak width is a balance between maximising the data from the peak, while minimising the influence of background and other features in the diffraction pattern. The fitted areas are illustrated by the vertical guides in Figure 5b,c.

Each $I_{\mathrm{rad}}\left(q\right)$ curve was pre‐filtered and baseline‐corrected prior to peak fitting. A robust spike filter suppressed isolated readout artefacts by comparing each sample to a local linear predictor built from neighboring points; samples that deviated by more than k median absolute deviations (MAD; k≈12) were temporarily excluded via:

(16) $$\begin{array}{cc} & |y_{i}-{\hat{y}}_{i}|\;\leq \;k\;\mathrm{MAD}\;\left(y-\hat{y}\right),\;{\hat{y}}_{i}\;\\ & =\;y_{i-1}+\alpha_{i}\;\left(y_{i+1}-y_{i-1}\right),\;\;\alpha_{i}=\frac{q_{i}-q_{i-1}}{q_{i+1}-q_{i-1}} \end{array}$$

A low‐order fitted background a+bq (or a constant baseline where indicated) was then estimated from the left/right edges of the selected q‐window and subtracted from the raw profile. A Savitzky–Golay smoother (odd window; polynomial order 2–3) was used only to initialise peak position and width; final parameter values came from parametric fits to the *unsmoothed*, baseline‐corrected data.

Peak positions were obtained by fitting a split pseudo‐Voigt line shape (allowing different left/right full widths at half maximum) on a shared linear background, using bounded, Poisson‐weighted robust least squares (soft‐$L_{1}$ loss). This follows the Thompson–Cox–Hastings pseudo‐Voigt formulation and its asymmetric/split variants widely used in powder diffraction.^[^ ^51^, ^52^, ^53^ ^]^ Poisson weights reflect counting statistics in Rietveld‐type least‐squares fits,^[^ ^54^, ^55^ ^]^ and the soft‐$L_{1}$ loss is a standard robust choice for peak fitting.^[^ ^56^ ^]^ With $q_{0}$ the peak centre, A the amplitude, η∈[0,1] the Lorentzian fraction, $G\left(u\right)=\exp\left(-4\ln2\;u^{2}\right)$ and $L\left(u\right)=1/\;\left(1+{\left(2u\right)}^{2}\right)$, the overall model therefore equated to:

(17) $$I\left(q\right)\;=\;a+bq\;+\;\left\{\begin{array}{cc} A\left[\eta \;L\;\left(\frac{q-q_{0}}{w_{L}}\right)+\left(1-\eta \right)\;G\;\left(\frac{q-q_{0}}{w_{L}}\right)\right], & q<q_{0},\\ A\left[\eta \;L\;\left(\frac{q-q_{0}}{w_{R}}\right)+\left(1-\eta \right)\;G\;\left(\frac{q-q_{0}}{w_{R}}\right)\right], & q\geq q_{0}, \end{array}\right.$$

with bounds $q_{0}\in \left[q_{\min},q_{\max}\right]$, $w_{L,R}\in \left[2\;\Delta q,\;0.95\left(q_{\max}-q_{\min}\right)\right]$ (here Δq is the median sampling step in q), and η∈[0,1]. Initial guesses for $\left(q_{0},A\right)$ were obtained from the smoothed profile via parabolic interpolation at the maximum and interpolation of the baseline‐corrected height. The initial FWHM came from the measured half‐height width; the initial η was estimated from the ratio of the 10% and 50% widths, $r=w_{10}/w_{50}$, using the Gaussian and Lorentzian limits $r_{G}=\sqrt{\ln10/\ln2}$ and $r_{L}=3$ duvh that:

(18) $$\eta_{0}\;=\;\mathrm{clip}\;\left(\frac{r-r_{G}}{\;r_{L}-r_{G}\;},\;0,\;1\right)$$

Suitable fitting was achieved for all fibers frames. The goodness‐of‐fit $\left(R^{2}\right)$, fitted background coefficients (a,b), left/right FWHM $\left(w_{L},w_{R}\right)$, and η were recorded along with the peak centre. The resulting estimates of lattice spacing were converted from reciprocal space via 14 where $q_{0}^{hkl}$ is the fitted centre for the {hkl} reflection and $d^{hkl}$ is reported in Å. Fixed settings for sector integration, pre‐filtering and fitting are summarized in **Table** **2**.

### 2.7 Lattice–Strain Mapping

Lattice strains for the graphitic {100} (axial) and {002} (radial) reflections were computed on a per‐frame basis from the d‐spacings extracted in Section 2.6. The specimen‐specific registration/interpolation hyperparameters actually used are shown in **Table** **3**. Briefly, i) a zero‐load reference field $d_{0}^{hkl}\left(x,y\right)$ is established at the raster coordinates, ii) align loaded rasters to the reference, iii) interpolate $d_{0}^{hkl}$ onto the loaded frame positions, and iv) form engineering lattice strains with tensile strain taken as positive (lattice expansion) and compressive as negative. Only frames labeled fibers by the {002}‐based classifier were processed; frames labeled resin/air were excluded from fibers‐specific metrics. The resulting strain estimates were detremined from:

(19) $$\varepsilon_{hkl}\;=\;\frac{d^{hkl}-d_{0}^{hkl}}{d_{0}^{hkl}},\;hkl\in \left\{100,002\right\}.$$

For each specimen, a dedicated zero‐load reference raster (0 N) was used to defined the unloaded lattice strain distribution $d_{0}^{hkl}\left(x,y\right)$.^[^ ^14^, ^33^ ^]^ This corresponds to the lattice strain distribution arising from the manufacturing process. From the corresponding diffraction patterns obtained from loaded samples, fibers frames with valid peak fits for both reflections were retained. Quality gates on the peak‐fit coefficient of determination were implemented to remove outliers such that $R_{\left\{002,100\right\}}^{2}\geq 0.95$. Let $\left(x_{i},y_{i}\right)$ denote the motor coordinates of the retained baseline frames and $d_{i}^{002},\;d_{i}^{100}$ their fitted spacings. The point set $\left\{\left(x_{i},y_{i},d_{i}^{hkl}\right)\right\}$ constitutes discrete samples of $d_{0}^{hkl}\left(x,y\right)$ which are required for the conversion of lattice strain estimates to strain via Equation (19).

Small stage drifts between rasters were corrected by estimating a global in‐plane translation (Δx,Δy) between the loaded raster and the baseline. For a given load step, let $\left({\tilde{x}}_{j},{\tilde{y}}_{j}\right)$ be the fibers‐frame coordinates measured at load. The median 1‐nearest‐neighbor (1‐NN) offset between $\left\{\left({\tilde{x}}_{j},{\tilde{y}}_{j}\right)\right\}$ and $\left\{\left(x_{i},y_{i}\right)\right\}$ was computed within a radius $r_{\mathrm{NN}}=\alpha \;h$, where h is the median fibers‐frame nearest‐neighbor spacing (effective grid step) of the 0 N raster and α is a dimensionless scale factor. The values of h and α used for each specimen are reported in Table 3 (such that h≈0.14--0.16mm and α≈0.9--1.1). The loaded coordinates were then translated as $x_{j}^{\mathrm{aln}}={\tilde{x}}_{j}+\Delta x$ and $y_{j}^{\mathrm{aln}}={\tilde{y}}_{j}+\Delta y$ and used for all subsequent interpolation and mapping. If the number of valid NN pairs fell below a small fractional threshold of the fibers set (e.g. <5% or <10 points), the conservative (Δx,Δy)=(0,0) was adopted and the step was flagged for QC.

The fact that baseline and loaded rasters are not identical grids means the zero‐load spacing at each loaded fibers position $\left(x_{j}^{\mathrm{aln}},y_{j}^{\mathrm{aln}}\right)$ needed to be obtained by IDW of the baseline fibers samples.^[^ ^57^, ^58^, ^59^ ^]^ This used up to k nearest neighbors within a search radius R=βh; the tuned k and β for each specimen are given in Table 3. With IDW power p, which is also listed in Table 3, the predictor reads:

(20) $$\begin{array}{cc} & d_{0}^{hkl}\;\left(x_{j}^{\mathrm{aln}},y_{j}^{\mathrm{aln}}\right)\;=\;\frac{\sum_{i\in N_{j}}w_{ij}\;d_{i}^{hkl}}{\sum_{i\in N_{j}}w_{ij}},\\ & \;w_{ij}\;=\;\frac{1}{{\left(\;∥\left[x_{j}^{\mathrm{aln}},y_{j}^{\mathrm{aln}}\right]-\left[x_{i},y_{i}\right]∥\;\right)}^{p}+\varepsilon } \end{array}$$

The hyperparameters (p,k,β) were chosen once per specimen by leave‐one‐out cross‐validation (LOO‐CV)^[^ ^60^, ^61^ ^]^ on the baseline raster in order to minimise the IDW prediction error expressed as microstrain, subject to a coverage constraint which is the fraction of baseline points successfully predicted, typically ≥0.97:

(21) $$\mu \varepsilon_{\mathrm{IDW}}^{hkl}\;=\;10^{6}\;{\mathrm{median}}_{i}\;\left(\frac{\left|\;d_{i,\mathrm{LOO}}^{hkl}-d_{i}^{hkl}\;\right|}{\mathrm{median}\;\left(d^{hkl}\right)}\right)$$

In practice, the tuned values (Table 3) fall within the stable ranges p∈[1.5,2.5], k∈[12,24], and β∈[1.6,2.8]; these values were then held fixed across all load steps for the corresponding specimen.

For each loaded fibers frame j, the axial and radial strains were formed directly at the aligned coordinates^[^ ^33^ ^]^ using the interpolated baseline values. In locations where a signed misalignment Δ was available (Section 2.5), per‐frame direction cosines were also stored for optional de‐mixing or regression. However no explicit rotation of the sector masks was applied during d‐spacing extraction (Section 2.6), i.e. the same fixed azimuthal sectors were used consistently across loads and specimens via:

(22) $$\varepsilon_{002,j}\;=\;\frac{d_{j}^{002}-d_{0,j}^{002}}{d_{0,j}^{002}},\;\varepsilon_{100,j}\;=\;\frac{d_{j}^{100}-d_{0,j}^{100}}{d_{0,j}^{100}}$$

Throughout, “axial” ($\varepsilon_{100}$) and “radial” ($\varepsilon_{002}$) denote fiber‐lattice directionsin turbostratic carbon, rather than macroscopic stress components. The stress used when reporting ε–stress slopes is the applied, global compressive engineering stress along the loading axis (warp), such that $\sigma_{\mathrm{app}}=\frac{L}{A}$ where L is the load recorded by the compression stage and A is the specimen's average cross‐sectional area obtained from XCT. Conversion from a strain–load slope $s_{L}=d\varepsilon /dL$ (in με/kN) to a strain–stress slope $s_{\sigma }=d\varepsilon /d\sigma$ (in με/MPa) uses $s_{\sigma }=s_{L}\left(\frac{A}{1000}\right)$ with L in kN and A in ${\mathrm{mm}}^{2}$. Reported slopes therefore represent apparent lattice compliances with respect to $\sigma_{\mathrm{app}}$.

At each load step, robust descriptors were computed over the valid fibers frames: the median ${\tilde{\varepsilon }}_{hkl}$ and a MAD scale,

(23) $$\begin{array}{cc} & {\hat{\sigma }}_{\mathrm{MAD}}\left(\varepsilon_{hkl}\right)\;=\;c_{N}\;\mathrm{median}\;\left(\;|\varepsilon_{hkl}-{\tilde{\varepsilon }}_{hkl}|\;\right),\\ & \;c_{N}\;=\;\frac{1}{\Phi^{-1}\left(0.75\right)}\;\approx \;1.4826 \end{array}$$

Here Φ is the standard normal cumulative distribution function and $\Phi^{-1}$ its quantile function. Since $\Phi^{-1}\left(0.75\right)=0.67448975$, the scaling $c_{N}=1/\Phi^{-1}\left(0.75\right)\approx 1.4826$ makes ${\hat{\sigma }}_{\mathrm{MAD}}$ consistent for σ under Gaussian errors.^[^ ^62^ ^]^

Load–strain slopes were estimated both by ordinary least squares with free intercept and by constrained fits through the origin. 95% confidence intervals for the through‐origin slope were obtained via bootstrap resampling at the *frame* level within each load, recomputing per‐load medians on each draw.

### 2.8 Orientation‐Aware Predictive Modeling and Validation

To encapsulate the atomic strain response of the material a powerful new analytical model has been developed. Although prior literature has focused extensively on the use of finite element methods to study wrinkles to great success,^[^ ^5^, ^14^ ^]^ the processing power, implicit assumptions and tailoring required to deliver these simulations can add additional barriers to widespread use by industrial partners. This gap has historically been filled by analytical models which have proven a powerful approach to provide high‐level predictions of performance from a given geometry.^[^ ^14^, ^63^ ^]^ However, with the advent of advanced regularisation‐based techniques which are able to better encapsulate fibers scale misorientation of fibers, more reliable and precise simulations can now be achieved, provided that the microscale response of a representative system is known. Therefore, in this study, a new physics‐anchored model has been established to build on this insight and provide an entirely new paradigm for predicting macroscale performance from NDT‐driven geometry of real parts.

This predictor builds on the prior stages of this paper via the link between local fibers orientation and lattice‐strain response in graphitic carbon fibers and laminated composites.^[^ ^33^, ^35^ ^]^ The predictor is cast as based around a physics driven, linear‐in‐parameters model. Robust estimation follows ridge‐regularized regression with a heavy‐tailed loss,^[^ ^64^, ^65^ ^]^ and model choice uses leave‐one‐scan‐out (group) cross‐validation at the raster level.^[^ ^66^ ^]^ An orientation‐aware, linear‐in‐parameters predictor was constructed for the per‐frame lattice strains $\varepsilon_{002}$ (radial) and $\varepsilon_{100}$ (axial), using the signed misalignment Δ. Slow in‐plane trends were represented by unitless 1D shape functions P=P(y) and W=W(x) rescaled to [0,1]; load enters as a scalar L (in N). The activated configuration uses the angle orientation channel, i.e. the misalignment in radians, and includes the first‐order interactions of orientation with P and W, as well as the average absolute strains at the nominal applied loads. For this process the misalignment term was converted to radians via:

(24) $$\gamma \;=\;\Delta \cdot \frac{\pi }{180}$$

Candidate base designs are load‐anchored families so that the prediction vanishes at zero load. For compactness, the base feature vectors are:

(25) $$\Phi_{\text{1D}}\;=\;{\left[\;L,\;L\;P\;\right]}^{⊤}$$

(26) $$\Phi_{\text{2D}}\;=\;{\left[\;L,\;L\;P,\;L\;W\;\right]}^{⊤}$$

(27) $$\Phi_{\text{2D}\_\text{INT}}\;=\;{\left[\;L,\;L\;P,\;L\;W,\;L\;P\;W\;\right]}^{⊤}$$

(28) $$\Phi_{\text{QUAD}}\;=\;{\left[\;L,\;L\;P,\;L\;W,\;L\;P^{2},\;L\;W^{2},\;L\;P\;W\;\right]}^{⊤}$$

(29) $$\Phi_{\text{L2}}\;=\;{\left[\;L,\;L\;P,\;L\;W,\;L^{2},\;L^{2}P,\;L^{2}W\;\right]}^{⊤}$$

Orientation terms activated are appended multiplicatively with load and the spatial shapes,

(30) $$\Phi_{\text{ori}}\;=\;{\left[\;L\;\gamma \;L\;P\;\gamma ,\;L\;W\;\gamma \;\right]}^{⊤}$$

and the full design for a given base family is

(31) $$\phi \;=\;\left[\begin{array}{c} \Phi_{\text{base}}\\ \Phi_{\text{ori}} \end{array}\right]$$

For each reflection hkl∈{002,100}, the prediction is linear such that,

(32) $${\hat{\varepsilon }}_{hkl}\;=\;\phi^{⊤}\;\beta^{\left(hkl\right)}$$

and satisfies the zero‐load anchoring by construction,

(33) $${\hat{\varepsilon }}_{hkl}\left(L=0\right)\;=\;0$$

Parameters are obtained via robust ridge regression with a Cauchy loss and an $\ell_{2}$ penalty such that τ≥0; no intercept is included:

(34) $${\hat{\beta }}^{\left(hkl\right)}\;=\;\arg\min_{\beta }\;\sum_{n=1}^{N}\rho \;\left(\phi_{n}^{⊤}\beta -\varepsilon_{n}^{\left(hkl\right)}\right)\;+\;\tau \;{∥\beta ∥}_{2}^{2}$$

where ρ(·) is the Cauchy penalty. Model selection was performed independently for $\varepsilon_{002}$ and $\varepsilon_{100}$ using leave‐one‐scan‐out cross‐validation at the scan (raster) level, comparing the families in Equations (25)–(29) over a ridge grid gives

(35) $$\tau \;\in \;\{\;0,\;10^{-6},\;10^{-4},\;10^{-3},\;10^{-2}\;\}$$

Selection is based primarily on cross‐validated root‐mean‐square error (RMSE), with the 95th percentile absolute error and mean absolute error as secondary criteria at comparable complexity. Calibration is summarized by a parity line between predictions and measurements in the held‐out scan such that,

(36) $${\hat{\varepsilon }}_{hkl}\;=\;a^{\left(hkl\right)}\;+\;b^{\left(hkl\right)}\;\varepsilon_{hkl}$$

where ideal calibration corresponds to $a^{\left(hkl\right)}\approx 0$ and $b^{\left(hkl\right)}\approx 1$. Map‐level validation was used to compare measured and predicted fields in absolute units on a common grid, with NaN‐aware smoothing used only for visual clarity and with non‐fibers regions masked consistently.

## 3 Results and Discussion

### 3.1 Fibers Detection and Non‐Fibers Masking

The azimuthal diffraction profiles arising from the integration process exhibited the characteristic two‐lobed texture in fibers‐bearing regions, enabling reliable binary separation of fibers from resin/air across all datasets. Applying the classification to each raster produced spatial masks with consistently high coverage in the region of interest, with median valid‐fibers fractions of 81.0% for the large‐wrinkle series, 85.0% for the small‐wrinkle series, and 92.9% for the pristine series. This preserved the dense support required for subsequent peak fitting and strain mapping and limited spurious contributions from texture‐poor areas.

In terms of fibers orientation the average deviation from $180^{\circ }$ was *0.5* 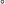 with an interquartile range of *0.5* 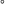. For the large wrinkled specimen the median deviation was *0.5* 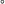 with the exception of the 1100 N terminal hold which was *1.0* 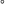. For the small wrinkle and pristine samples the average deviation was *0.5* 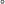, with rare pre‐fit outliers reaching at most *8.5* 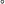. This behavior is consistent with the expected the fibers texture and indicates that residual background removal and spike suppression were sufficient to avoid false multi‐peak detections.

**Figure 7:**
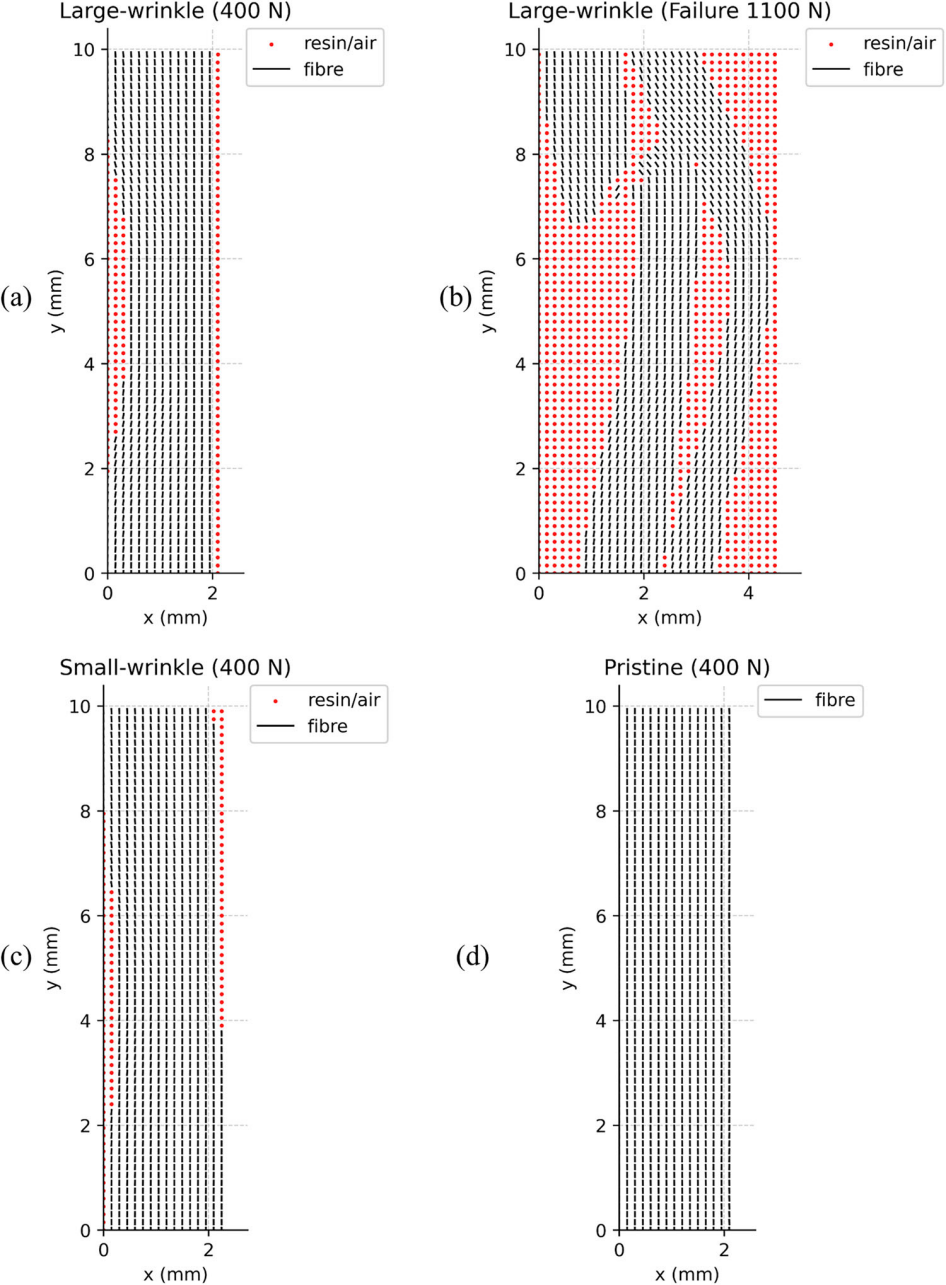
Composite fibers‐orientation map: Black lines indicate the local fibers orientation (δ and red dots denote resin/air. a) Large‐wrinkle (400 N), b) Large‐wrinkle–Failure at 1100 N, c) Small‐wrinkle (400 N), d) Pristine (400 N).

Spatially, the fibers masks closely followed the laminate footprint, with only sparse holes (**Figure** **7**), resulting in contiguous domains suitable for map‐based statistical analysis. In specimens with wrinkles, low‐density regions were more frequent near steep geometric transitions adjacent to the wrinkle. Nevertheless, coverage in these areas remained sufficient for capturing local trends. The pristine specimen exhibited the most uniform mask, consistent with stable two‐lobe profiles across the field of view. Importantly, all subsequent d‐spacing fits and strain calculations were confined to the fibers masks. This approach avoids over‐smoothing or introducing artefacts in regions lacking crystallographic texture, and ensures that coverage metrics reported later reflect only fibers‐supported estimates.

Robustness checks indicated limited sensitivity of this fitting process. Varying the smoothing and baseline settings led to at most *2%* changes in orientation. This suggest that the qualitative conclusions drawn from the masks are insensitive to moderate perturbations of analysis configuration.

### 3.2 Peak Fitting Performance and Quality Control

Across all loads and specimens, the pipeline converged robustly for both {002} and {100}. No fits were rejected by QC for either reflection at any load. This outcome indicates that the fibers‐only classification stage effectively excluded texture‐poor regions and that the split pseudo‐Voigt model provided a stable description of the diffracted intensity, including at the terminal hold of the large‐wrinkle series. From this starting point, centre‐position uncertainties from the fit covariance were propagated from q to d via d=2π/q to report σ(d) and σ(d)/d. An overview of each load, number of frames and median σ(d) for both orientations is given in **Table** **4**.

**Table 4:** Peak‐fit uncertainty after QC in fibers‐masked regions (per specimen and load). All fits passed QC (100% acceptance; 0% rejection) at every condition.

Specimen	Load [N]	Fibers frames	Median $\sigma (d_{002})$ [$\times 10^{-4}d$]	Median $\sigma (d_{100})$ [$\times 10^{-4}d$]
*Large‐wrinkle*				
	0	862	1.70	2.05
	200	838	1.71	2.07
	400	841	1.73	2.09
	600	843	1.74	2.12
	800	858	1.78	2.16
	1100 (Failure)	1115	1.81	2.20
*Small‐wrinkle*				
	0	937	1.68	2.02
	400	947	1.71	2.07
	800	939	1.74	2.11
	1200	941	1.76	2.15
*Pristine*				
	0	1023	1.62	1.98
	400	1005	1.63	2.01
	800	1005	1.65	2.03
	1200	1030	1.66	2.06

Uncertainty on peak centre, expressed as $\sigma \left(d\right)/d\times 10^{-4}$, remained both low and load‐stable for all samples and peaks. For the {002} peak, the uncertainty had a median of 1.72 with an interquartile range (IQR) of 1.35–2.05 and for the {100} peak, the median uncertainty was 2.08 (IQR 1.68–2.60). It is important to note that sampling density in unstrained lattice parameter maps is governed solely by the mask rather than by fitting or QC. This means these narrow parameter spreads and the absence of rejected peaks provide strong support for the microstrain maps and the load‐dependent profiles across the wrinkle, limiting susceptibility to artefacts from texture‐poor areas.

### 3.3 Absolute d‐Spacing Maps ($d_{100}$, $d_{002}$)

In order to visualise the distribution of d‐spacing, heat maps for each specimen and load established. A consistent color bar scale is used based on the maximum range of d‐spacing values observed, which are:

(37) $$d_{100}\in \left[2.075\;Å,\;2.090\;Å\right],\;d_{002}\in \left[3.515\;Å,\;3.546\;Å\right]$$

**Figure 8:**
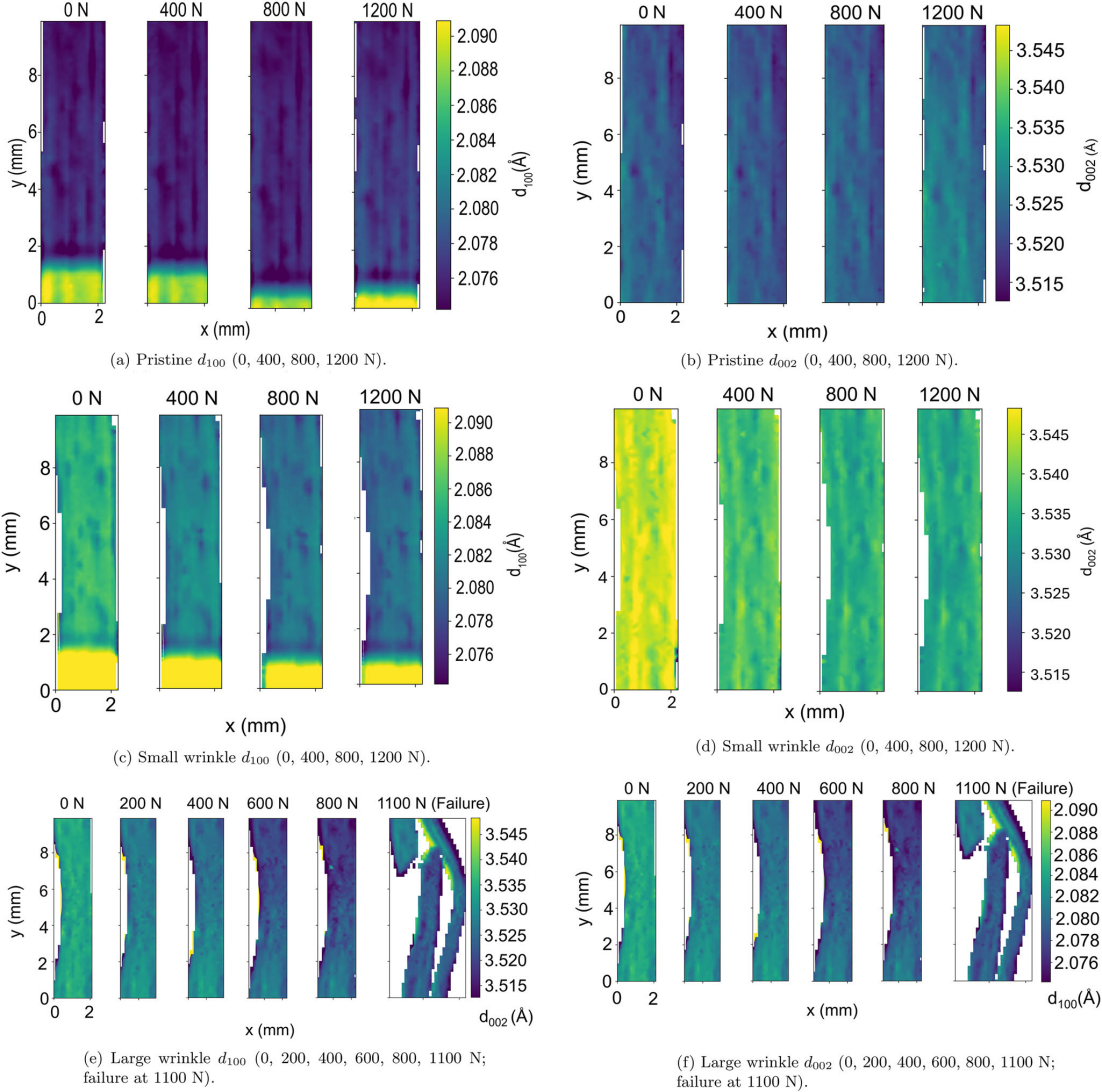
Absolute d‐spacing maps. **Rows**: pristine (top), small wrinkle (middle), large wrinkle (bottom). **Columns**: $d_{100}$ (left, axial) and $d_{002}$ (right, radial). fibers‐only mask; common color scale used for each peak.

Maps for the large‐wrinkle coupon show a progressive darkening (lattice contraction) with load and, at 1100N, the field fragments into diagonally banded domains consistent with the geometry change induced after failure. Small‐wrinkle coupons contract steadily but retain spatial continuity up to 1200N. Pristine coupons remain the most uniform; away from the end‐affected bright band, loaded fields lie close to the mid–lower part of the color scale. Representative maps are provided in **Figure** **8**. In the case of the {100} lattice spacing, the bottom of the sample was influenced by shadowing and should be interpreted as boundary‐condition effects rather than wrinkle signatures.

**Figure 9:**
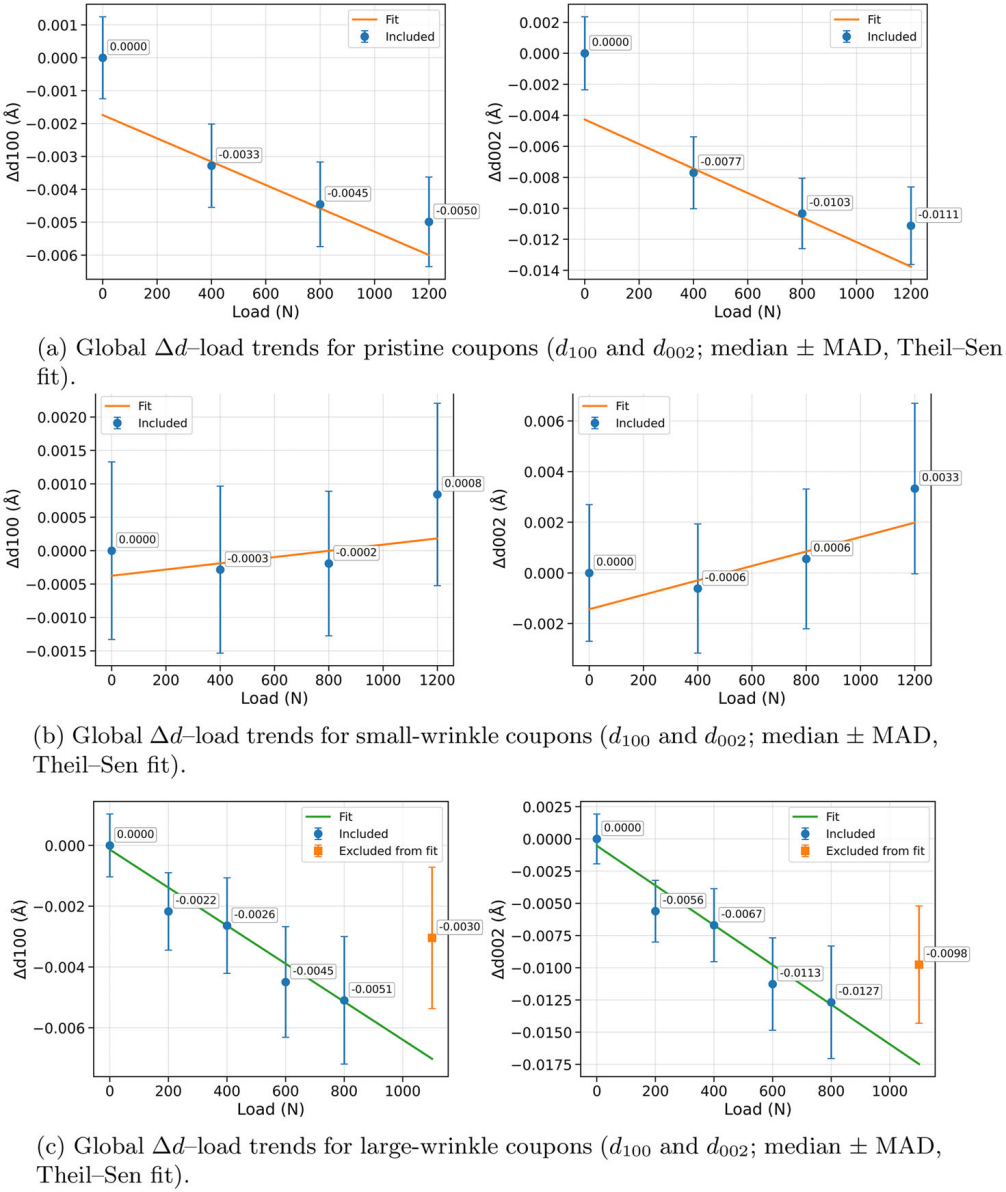
Specimen‐wise global trends of Δd as a function of load for {100} (axial) and {002} (radial) reflections. Each point represents the global median within the fibers mask, with vertical bars showing the MAD. Theil–Sen lines provide robust estimates of slope during loading prior to failure.

To summarise these point measurements into compact a reviewer‐friendly metric the values of

(38) $$\Delta d_{hkl}\left(L\right)\;=\;{\tilde{d}}_{hkl}\left(L\right)-{\tilde{d}}_{hkl,0}$$

where ${\tilde{d}}_{hkl}\left(L\right)$ is the *spatial median* of the fitted d–spacings over the fibers‐only mask at load L, and ${\tilde{d}}_{hkl,0}\equiv {\tilde{d}}_{hkl}\left(0\right)$ is the corresponding median at the unloaded (0 N) reference for that specimen (i.e. the specimen‐wise “unstrained” lattice parameter used as the reference). The median of these values along with the median absolute deviation (MAD) were determined for visualisation as shown in Figure **9**. A Theil–Sen line was fitted to this series to give a robust estimate of the gradient in Å/N. The terminal 1100N step of the large‐wrinkle series (post‐failure) was excluded from the fit. Figure 9 plots the relative variation in d to enhance visibility of subtle structural variations. When fitted against the original d‐values, the deviations appear minimal and below the perceptual threshold of the plot, resulting in fits that appear indistinguishable from the data. Therefore, the choice to plot Δd was intentionally selected to resolve fine differences between specimens. However, it should be noted that this artificially amplifies the apparent magnitude of the error bars (which are typically less than 0.05%) as well as the fitting error.

For the large‐wrinkle coupon the global medians show a monotonic decrease in $\Delta d_{100}$ with a slope of $-6.26\times 10^{-6}\;ÅN$ ^−1^, while a stronger decrease is observed for $\Delta d_{002}$ with slope $-1.54\times 10^{-5}\;ÅN$ ^−1^. Radial ({002}) contraction is therefore ≈ 2.5× the axial ({100}) contraction, indicating stronger inter‐layer sensitivity to compression. After failure, the 1100N medians for {100} and {002}) fall off the fitted trend as the failure unloads large sections of the specimen.

For the small‐wrinkle coupon, the slopes are $-3.55\times 10^{-6}\;ÅN$ ^−1^ for $\Delta d_{100}$ and $-7.91\times 10^{-6}\;ÅN$ ^−1^ for $\Delta d_{002}$. This demonstrates a similar relationship between contraction in the {002} than in {100} by a factor of ≈ 2.2×.

For the pristine coupon, trends are flat within the comparatively large MAD bars: $\Delta d_{100}$ has a slight positive slope of $+4.65\times 10^{-7}\;ÅN$ ^−1^ and $\Delta d_{002}$ has $+2.85\times 10^{-6}\;ÅN$ ^−1^. These slight positives likely reflect the shadow‐affected bright band and subtle load‐dependent changes in fibers‐mask coverage rather than being representative of the lattice strain response of the material.

In summary, the maps 8 reveal where the lattice changes, whereas the global trends 9 in quantify how much on average the lattice shifts with load. The combined picture is internally consistent: strong, monotonic negative Δd slopes for wrinkled coupons; near‐zero global shifts for pristine; and a systematic ratio of ≈2–2.5 between {002} between the {100} responses.

### 3.4 $\varepsilon_{100}$ and $\varepsilon_{002}$: Lattice‐Strain Maps and Global Trends

After determining robust {100} and {002} peak positions at every fibers‐bearing raster point, lattice strains were computed point‐to‐point against an unloaded 0N baseline so that each loaded map uses the local $d_{0}\left(x,y\right)$ from the same physical location. Small inter‐scan drifts were removed with a global in‐plane translation derived from nearest‐neighbor offsets; $d_{0}$ was then interpolated onto the loaded grid to allow strain to be quantified via Equation 19.

**Figure 10:**
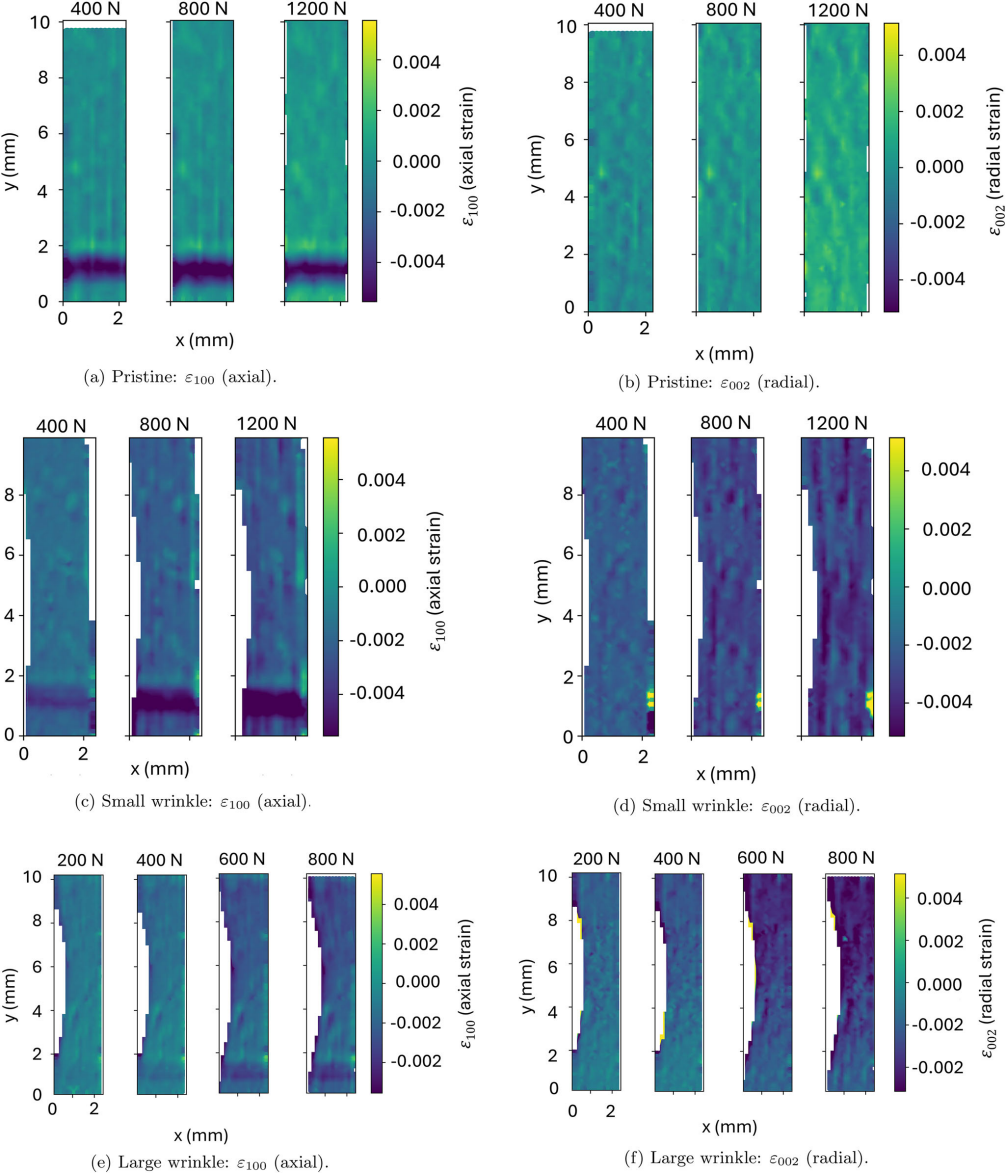
Point‐wise lattice‐strain heatmaps for axial $\varepsilon_{100}$ (left) and radial $\varepsilon_{002}$ (right), ordered as **pristine** → **small wrinkle** → **large wrinkle**. Axes in mm; common color scales are used in all panels.

As shown in **Figure** **10**, across all specimens the strain maps retain the spatial continuity of the lattice spacing. In the case of the {100} strain, the bottom of the sample was influenced by shadowing and should be interpreted as boundary‐condition effects rather than wrinkle signatures. In both wrinkled coupons, strains are more compressive close to the wrinkle and along its flanks, with magnitude increasing monotonically with load. Localisation is strongest in $\varepsilon_{002}$ and, for the large‐wrinkle specimen, evolves by 800N into a broad compressive corridor. The pristine coupon remains near‐uniform.

**Figure 11:**
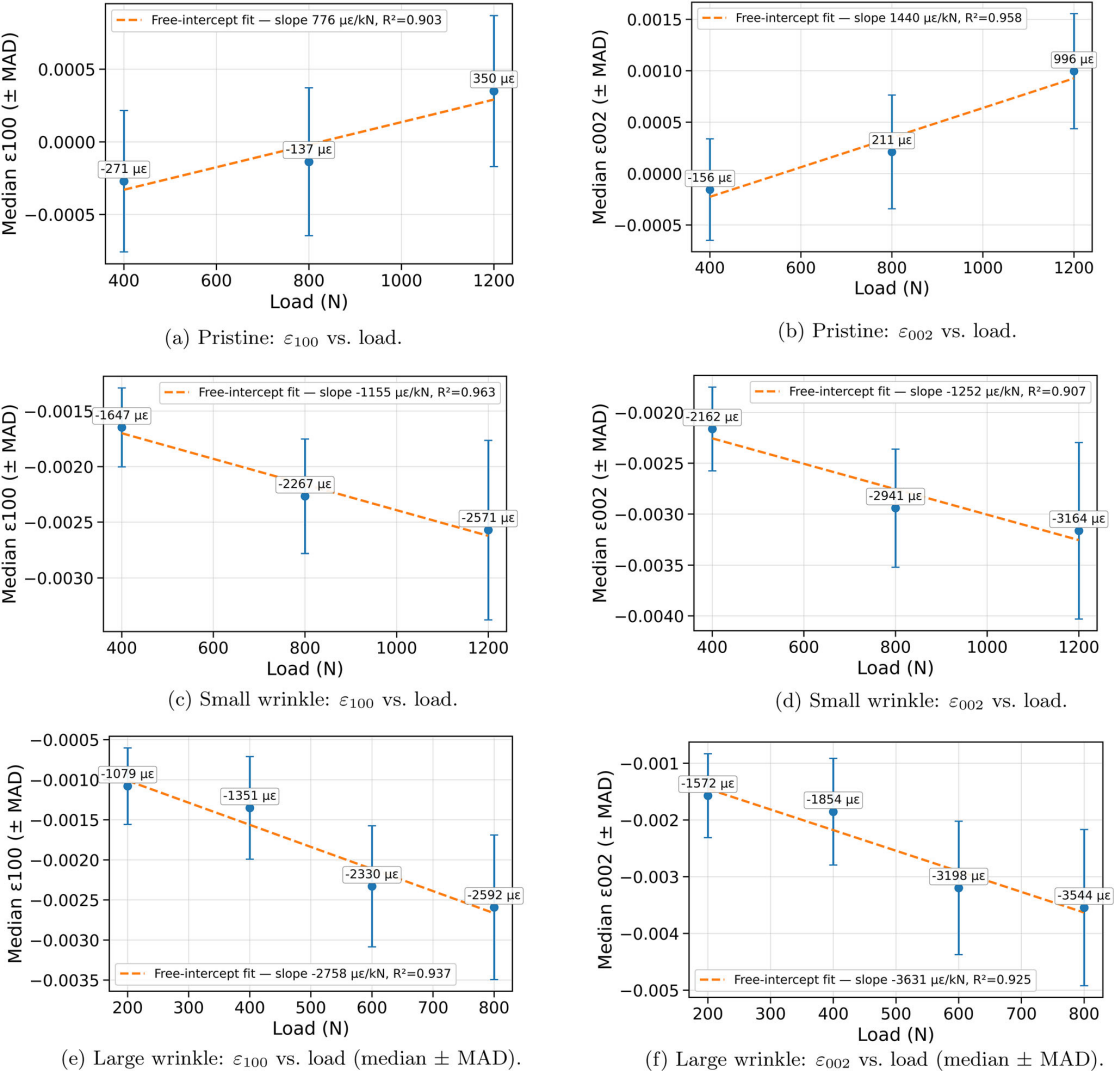
Median ε–load relations (points: median, bars: MAD; dashed line: free‐intercept fit). Rows: pristine, small wrinkle, large wrinkle. Columns: $\varepsilon_{100}$ (axial, left) and $\varepsilon_{002}$ (radial, right).

To provide insight into the average trends in strain, the median strain was plotted against load (error bars: MAD), and fitted using a linear model. The resulting trends are summarized in **Figure** **11**, which shows relative strain such that the unloaded estimate is 0 in all cases. It should also be noted that registration between the unloaded sample and large wrinkle specimen after failure is not possible, and therefore, this point is excluded from this analysis. Slopes are reported in με/kN along with the confidence of fit $R^{2}$.

To compare coupons with different cross‐sections, the ε‐load slopes (με/kN) were converted to ε‐stress slopes (με/MPa). These slopes are reported in με/MPa which is a relative measure of apparent lattice compliance. It is important to highlight that the length scales and locations of measure within this calculation are distinct; the strain refers to atomic‐scale lattice strain within the fibers, and the stress refers to the average macroscale stress across the entire sample (both the fibers and matrix). In the case of the $\varepsilon_{002}$ plots this also corresponds to two distinct directions; the load is applied along the fibers axis and the strain is measured perpendicular to this direction, meaning that Poisson effects are also integrated into this expression. Therefore these numbers need to be carefully contextualized and not compared to other indpendent methods without further processing, they are however, useful for relative comparisons in the data interpretation for this particular study.

Using this approach the gradients in Figure 11 can be quantified. The large‐wrinkle specimen exhibits negative strain gradients, with $\varepsilon_{002}$ and $\varepsilon_{100}$ slopes of −3631 and −2758με/kN, respectively. In the small‐wrinkle specimen, the gradients are shallower but remain negative: $\varepsilon_{002}$ at −1252με/kN and $\varepsilon_{100}$ at −1155με/kN. By contrast, the pristine specimen shows modest positive gradients, with $\varepsilon_{002}$ and $\varepsilon_{100}$ slopes of +1440 and +776με/kN, respectively, consistent with near‐uniform strain fields in the absence of wrinkle‐induced localisation.

The overall ranking established from Δd (wrinkled ≫ pristine; radial > axial; large‐wrinkle ≥ small‐wrinkle) is reproduced by the ε–load slopes. This alignment is expected because ε combines the unloaded lattice parameter with load‐induced changes in d.

Using a single fitting rule for all series clarifies comparability across coupons. The median ε–stress slopes (apparent lattice compliance) are ≈ −14.5με/MPa (002) and −11.0με/MPa (100) for the large‐wrinkle specimen; −5.0με/MPa (002) and −4.6με/MPa (100) for the small‐wrinkle specimen; and near‐zero to weakly positive for the pristine specimen. The strains are referenced point‐wise to the local 0N raster on a common grid, therefore the fields can be overlaid directly on XCT, providing a practical bridge from crystallographic response to the locations where damage initiates.

### 3.5 Strain Localization and Damage Precursors

**Figure 12:**
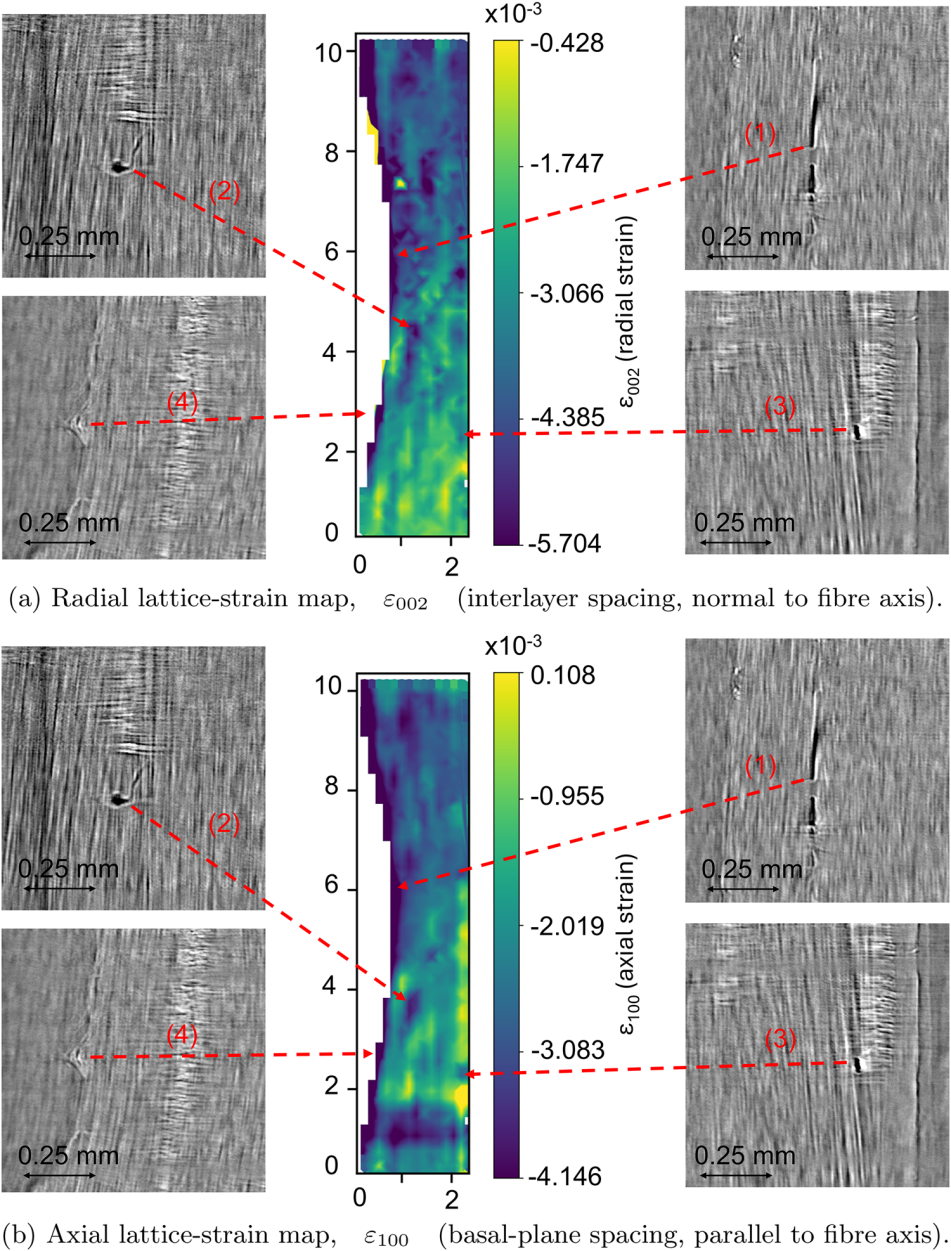
Co‐located XCT sections and lattice‐strain maps at 800 N (large‐wrinkle coupon). In each panel, the central color map shows the WAXS‐derived strain field; surrounding panels show representative XCT sections taken at the locations indicated by dashed guides. Red callouts denote: 1) delamination/matrix cracking, 2) fibers kink band, 3) delamination/matrix‐crack formation, and 4) local wrinkle bulge. Scale bars: 0.25 mm.

One of the most powerful aspects of combining XCT with WAXS methods is the ability to analyse strain localisation prior to failure. In this study, this is particularly dominant in the large wrinkled specimen which was scanned prior to failure at 800 N. **Figure** **12**a,b shows the diffraction‐derived lattice strain fields at 800 N at this load along with the XCT sections taken from the same region of interest. The radial fibers strain $\varepsilon_{002}$ (interlayer spacing, normal to the fibers axis) is shown in Figure 12a. The axial fibers strain $\varepsilon_{100}$, corresponding to basel‐plane spacing (parallel to the fibers axis) is shown in Figure 12b. This load precedes the terminal hold at 1100 N where failure was detected by the load stage and the sample held stationary. The CT slices in Figure 12 represent 2D sections of the sample, capturing fibers ply orientations and associated defects. These features lie near the resolution limit of the B16 beamline configuration, where the flexibility required for sequential CT and WAXS acquisition imposes constraints on achievable image quality. While individual fibers cannot be resolved, importantly the system provides sufficient resolution to identify microscale structural artefacts and correlate them with strain localisations observed in the WAXS data.

The two strain maps consistently reveal a compressive corridor straddling the wrinkle, with the most compressive values concentrated along its flanks rather than directly at the geometric crest. Regions labeled (1) coincide with locally high compressive microstrain in both $\varepsilon_{100}$ and $\varepsilon_{002}$ and match XCT evidence of delamination and matrix cracking; the alignment of these hot–spots across modalities indicates that through–thickness constraint generated by the wrinkle drives interlaminar tension and shear, which are relieved by crack opening between plies. Feature (2) corresponds to a fibers kink band that forms within the same corridor of elevated compression, while (3) marks a zone of incipient delamination/matrix–crack formation toward the specimen end. Feature (4) identifies the local wrinkle bulge, where sharp strain gradients surround the geometric perturbation.

Quantitatively, the maps at 800 N are consistent with the load trends reported earlier: for the large–wrinkle coupon the median radial strain is more compressive than the axial counterpart (typical medians at 800 N $\approx -3.54\times 10^{-3}$ for $\varepsilon_{002}$ and $\approx -2.59\times 10^{-3}$ for $\varepsilon_{100}$), reflecting stronger contraction of the interlayer spacing than of the basal–plane spacing under compression. The corresponding median slopes obtained from the full series up to 800 N were −3631με/kN for $\varepsilon_{002}$ and −2758με/kN for $\varepsilon_{100}$, confirming the systematic radial > axial sensitivity at the wrinkle.

The XCT panels corroborate the diffraction results: delamination and matrix cracks nucleate where the radial microstrain is most compressive, and the kink band aligns with the compressive corridor seen in the strain maps. To quantify the relationship between microstrain and failure mechanisms, a short correlation analysis was performed using the XCT–assisted WAXS dataset at 800 N. In the large‐wrinkle specimen, regions exhibiting the most compressive radial lattice strain $\varepsilon_{002}$ consistently aligned with damage features observed in XCT, including delamination, matrix cracking, and fibers kink banding (Figure 12). These regions exhibited $\varepsilon_{002}$ values approaching $-3.5\times 10^{-3}$, while adjacent undamaged zones remained near $-1.5\times 10^{-3}$. This contrast suggests a threshold‐like behavior, where radial microstrain exceeding a critical value correlates with the onset of irreversible damage. The axial strain $\varepsilon_{100}$ also showed localisation, but with a weaker gradient and less spatial specificity. This supports the interpretation that $\varepsilon_{002}$ is a more sensitive indicator of through‐thickness constraint and interlaminar stress, consistent with prior studies on wrinkle‐induced failure in CFRP.^[^ ^14^ ^]^ The predictive model reproduces these strain corridors with sub‐millistrain accuracy, confirming that the spatial distribution of microstrain is not only measurable but also predictable from wrinkle geometry and fibers orientation. These findings suggest that lattice strain mapping, particularly in the radial direction, can serve as a quantitative precursor to damage, enabling early intervention and refinement of rejection criteria in composite inspection workflows. This mirrors the absolute d–spacing behavior, where fields progressively decrease with load and then fragment at 1100 N, consistent with a geometry change at failure. By construction, the present strain maps share the same coordinate frame as XCT, so the spatial correspondence between (1) and (4) and the microstrain hot–spots is preserved without manual warping or subjective interpretation.

Mechanistically, these observations support a wrinkle‐mediated failure sequence in hybrid carbon/glass laminates under compression. Local fibers misalignment around the bulge concentrates through–thickness constraint; the radial response of the graphitic interlayers ($\varepsilon_{002}$) is therefore the earliest and strongest indicator of damage‐prone regions, followed by axial compression that facilitates microbuckling and kink band formation. The co–evolution of $\varepsilon_{002}$ and $\varepsilon_{100}$ provides a crystallographic signature for the transition from reversible lattice contraction to irreversible damage, with XCT supplying the direct images of delamination and cracking as these thresholds are crossed.

The signed misorientation term Δ, derived from the azimuthal texture of the {002} reflection, quantifies the local deviation of the fibers axis from the nominal loading direction. This deviation is a direct consequence of wrinkle geometry, which induces out‐of‐plane fibers undulation and alters the local stress transfer pathways within the laminate. In regions of elevated Δ, the fibers–matrix interface experiences increased shear and interlaminar tension, which in turn modulates the lattice strain response. The coupling between Δ and microstrain reflects the mechanical constraint imposed by the wrinkle: as fibers rotate away from the load axis, the interlayer spacing contracts more rapidly under compression, producing stronger $\varepsilon_{002}$ gradients. These gradients are spatially aligned with damage precursors such as delamination and kink banding, as shown in Figure 12. The orientation‐aware predictor incorporates Δ as a key input, enabling the model to reproduce the asymmetric strain corridors observed experimentally. This behavior is consistent with prior studies on fibers misalignment and strain localisation in wrinkled CFRP laminates,^[^ ^14^, ^33^ ^]^ and supports the interpretation of Δ as a geometric driver of microstrain amplification and damage onset.

This joint XCT‐WAXS perspective shows that lattice–strain localisation around a wrinkle is predictive of where delamination and kink bands appear, several load steps before catastrophic failure. In practice, this suggests that radial microstrain gradients ($\varepsilon_{002}$) can act as early non–destructive indicators of wrinkle severity, while axial microstrain ($\varepsilon_{100}$) helps discriminate zones predisposed to fibers kinking. Embedding such metrics within inspection protocols would improve triage of wrinkled regions in aerospace relevant laminates. This would also underpin the orientation–aware predictive model, which was shown to effectively reproduce the measured strain fields across the various load steps applied in this study.

While the present study was conducted at a synchrotron facility, the core principles of the XCT–WAXS methodology are transferable to laboratory and industrial inspection environments. Laboratory‐based WAXS systems equipped with microfocus sources and motorized raster stages can replicate the diffraction mapping protocol used here, albeit with longer acquisition times and, typically, reduced detector resolution. This may influence measurement precision and limit applicability for in situ assessment due to the viscoelastic nature of CFRPs, however this type of analysis remains viable for studying the influence of production on strain distribution generated. Similarly, industrial XCT systems can provide sufficient geometric context to guide targeted diffraction scans. The alignment procedure between XCT and WAXS datasets employed in this study and raster alignment, is compatible with such systems and does not rely on synchrotron‐specific infrastructure. Recent advances in compact WAXS instrumentation and automated data reduction pipelines further support the feasibility of deploying this approach in production or maintenance settings.^[^ ^14^, ^34^ ^]^ Although laboratory systems may not match the spatial resolution or throughput of synchrotron beamlines, the ability to correlate strain fields with geometric features remains intact. This opens a pathway for integrating crystallographic strain mapping into non‐destructive evaluation workflows, enabling early detection of damage‐prone regions in composite components. In particular, the identification of radial microstrain gradients ($\varepsilon_{002}$) as precursors to delamination and kink banding after production of a part, suggests that targeted WAXS scans informed by XCT could enhance triage and reduce false positives in defect assessment.

### 3.6 Model–Measurement Comparison

Having established at 800 N that strain localisation flanks the wrinkle and coincides with XCT evidence of delamination and kink banding, attention is now turned to the orientation‐aware predictor from Section 2.7. The question here is whether a compact, load‐anchored model using spatial shape functions and the signed misalignment Δ can reproduce the measured fields across load steps for the large‐wrinkle coupon, and whether it captures the stronger radial response ($\varepsilon_{002}$) relative to the axial ($\varepsilon_{100}$) identified earlier. Acquisition loads, masking and per‐point strain formation follow the protocol already detailed, including the 0 N co‐registered 0 N reference and fibers‐only processing.

**Figure 13:**
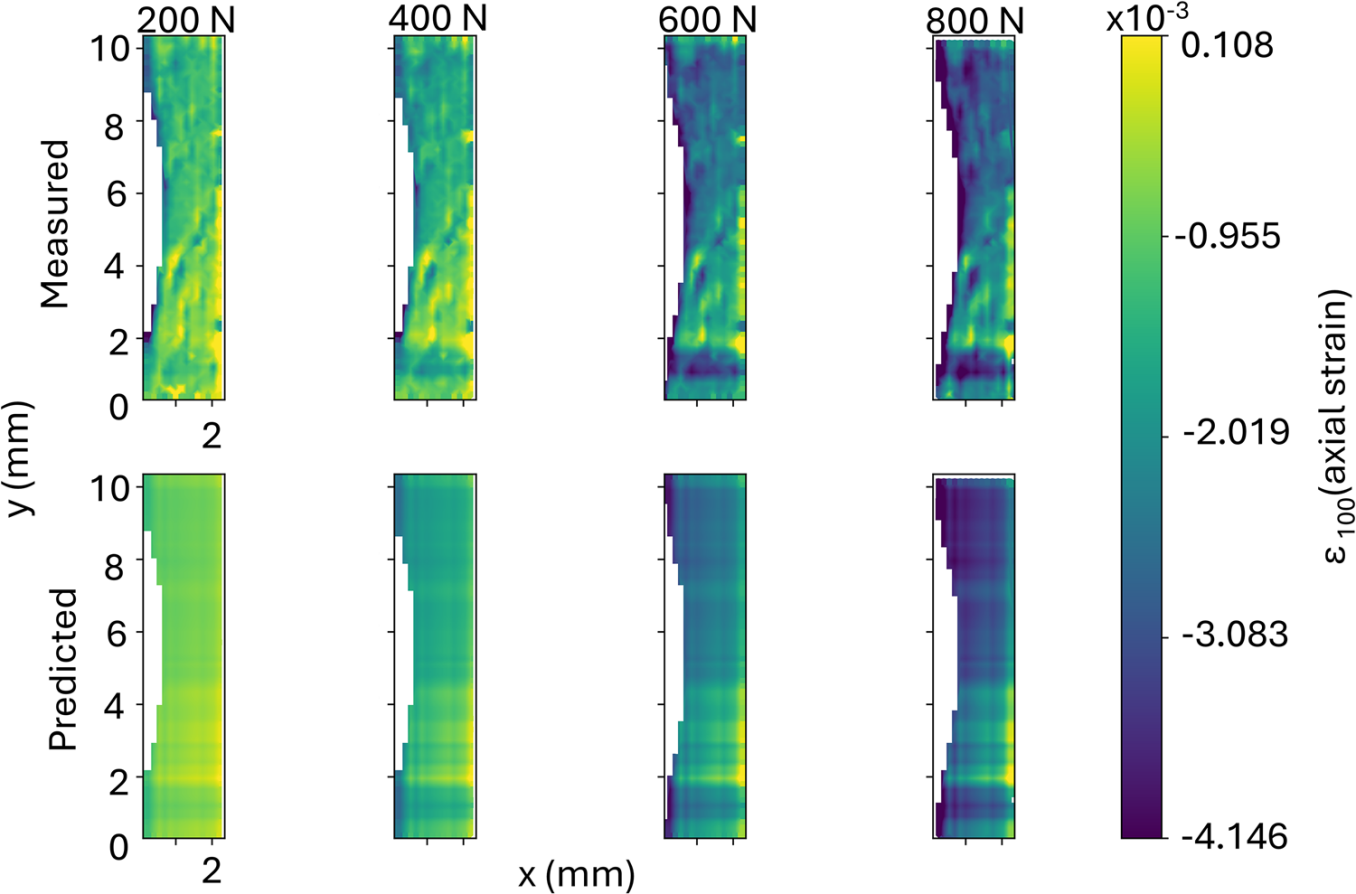
Large‐wrinkle coupon: axial lattice strain $\varepsilon_{100}$ (measured, top row) versus model prediction (bottom row) at 200, 400, 600, and 800 N. Common color scale as in earlier panels.

The axial comparison in **Figure** **13** shows that the predictor reproduces the broad compressive corridor flanking the wrinkle and its monotonic growth with load. Large‐scale gradients are well captured, while very fine, high‐wavenumber features in the measurements appear softened in the prediction. This is an expected consequence of the model's low‐order spatial basis and the deliberate regularisation used in § 2.7. Crucially, the map‐level discrepancy aggregated over the fibers mask at the 800 N hold remains sub‐millistrain: for $\varepsilon_{100}$, the mean absolute error (MAE) is $∼4.7\times 10^{-4}$ and the root‐mean‐square error (RMSE) is $∼6.6\times 10^{-4}$, consistent with the load‐dependent median trends reported for the large‐wrinkle specimen.

**Figure 14:**
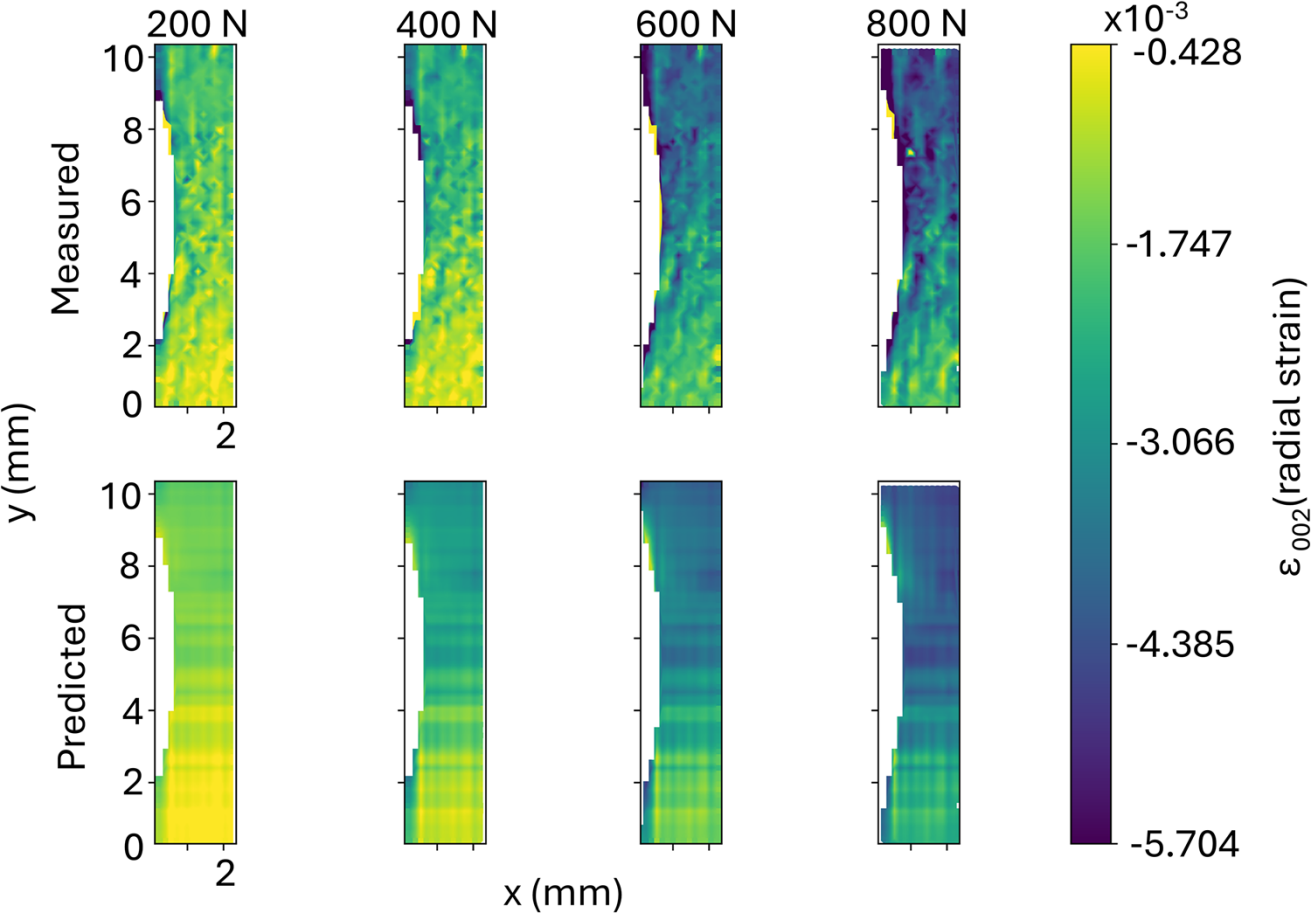
Large‐wrinkle coupon: radial lattice strain $\varepsilon_{002}$ (measured, top row) versus model prediction (bottom row) at 200, 400, 600 and 800 N. The model captures the stronger radial contraction and the development of the compressive corridor adjacent to the wrinkle.

The radial comparison in **Figure** **14** confirms the increased sensitivity of the interlayer spacing under compression. The corridor is deeper and spreads with load in both measurement and prediction. At 800 N, the corresponding error summary across the fibers mask is RMSE $\approx 8.6\times 10^{-4}$ and MAE $\approx 5.3\times 10^{-4}$ for $\varepsilon_{002}$. These errors are small relative and they respect the established ranking that the radial response exceeds the axial one for the large‐wrinkle coupon.

**Figure 15:**
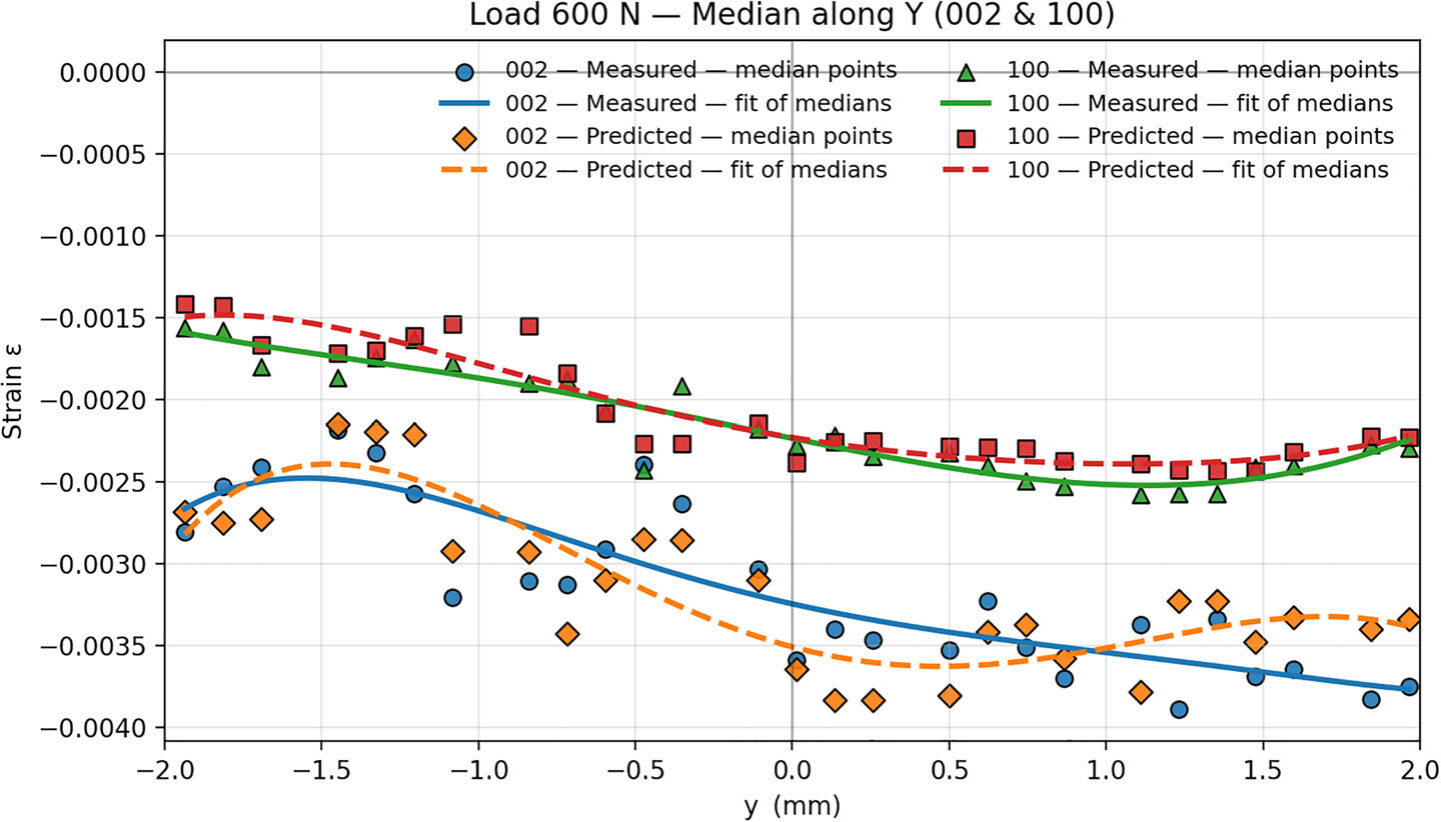
Large‐wrinkle coupon at 600 N: median $\varepsilon_{100}$ and $\varepsilon_{002}$ in the transverse direction (y, ±2 mm about the wrinkle centre at y=0). Symbols are slice medians; lines are smooth fits to those medians for measured and predicted fields.

In order to better visualise the correlation between the model and experimental results a section‐wise view was developed. **Figure** **15** plots the median profiles across y∈[−2,2] mm at 600 N, with y=0 at the wrinkle centre. The 600 N case was selected for section‐wise comparison as it represents the highest load prior to the onset of pre‐failure artefacts, which begin to emerge at 800 N as indicated by the increase in MAD in Figure 11e and 11f. The predictive model is designed to capture elastic strain evolution and does not account for damage mechanisms; therefore, 600 N provides the most reliable basis for direct comparison between experimental and simulated strain fields. Both reflections show that the predicted curves follow the measured medians closely in phase and overall amplitude, reproducing the asymmetric troughs that mark the compressive corridor. Residuals are largest at the most extreme compressive pockets and near the specimen edges, consistent with the predictor's low‐order design and with end‐affected bands noted previously. Nevertheless, the agreement across the central ≈±1.5 mm region, which contains the wrinkle footprint, confirms that the model preserves the correct ranking (radial more compressive than axial) and the spatial placement of peak compression around the bulge.

Taken together, the maps, the quantitative errors at 800 N, and the transverse medians indicate that a lightweight, orientation‐aware predictor can recover the dominant evolution of microstrain around a manufacturing wrinkle while remaining anchored to zero load and robust to modest drift between rasters. In practical terms, this suggests a route to computationally inexpensive a priori field estimates that agree with measured WAXS maps at the level needed for triage. The model highlights the same corridors where $\varepsilon_{002}$ becomes most negative, i.e. the locations subsequently implicated in delamination and kink banding in Section3.5, and distinguishes these from benign weave texture. Embedding such predictors alongside XCT in inspection workflows could shorten time‐to‐decision and focus confirmatory diffraction on the most critical sub‐regions of aerospace‐relevant laminates.

Coupons isolate the local response of a single wrinkle under quasi‐static compression with well‐defined load control and a short gauge ($2\times 2\;{\mathrm{mm}}^{2}$ section, 25mm length), suppressing global buckling while permitting local kinking. In large segments, the same wrinkle resides in a distinct constraint environment: far‐field stiffness, ply‐to‐ply continuity, curvature, and local attachments can drive subregions closer to strain control. These boundary conditions alter the amplitude and spatial reach of the compressive corridor but not its qualitative signature: $\varepsilon_{002}$ localizes on the wrinkle flanks, precedes delamination and matrix cracking, and co‐localises with kink‐band onset. Accordingly, $\varepsilon_{002}$ gradients constitute transferable early indicators of damage‐prone zones, whereas absolute levels and corridor extent should be calibrated to each component architecture. Consistency with the present dataset is evidenced by XCT‐assisted WAXS at 800N, where radial microstrain peaks align with delamination and a kink band (Figures 12), and by the systematic radial>axial ranking across loads (Figures 8, 9, 10, 11).

## 4 Conclusion 

The present study establishes a new XCT‐assisted synchrotron WAXS methodology for diagnosing degradation in carbon‐fibers laminates. Using a single instrument configuration, rastered WAXS delivered point‐wise maps of fibers orientation and lattice strain, while full‐field XCT provided imaging. A fully automated reduction pipeline provided microstrain‐resolved fields that overlay directly with the tomograms to give quantitative insight into the radial ($\varepsilon_{002}$) and axial ($\varepsilon_{100}$) responses.

### 4.1 Key findings

i) **Reliable crystallographic sensing**. The fiberes/no‐fibers classifier operated robustly across loads and specimens; orientation quantification showed a median deviation of $\approx 0.5^{\circ }$. All {002} and {100} peaks were accepted (100% fit acceptance), with tight peak‐centre uncertainties $\sigma \left(d\right)/d∼\left(1.7\text{--}2.1\right)\times 10^{-4}$, enabling robust microstrain quantification.
ii) **Load‐dependent lattice response with radial amplification**. Wrinkled specimens compressed monotonically in both reflections, with the radial response exceeding the axial. For the large‐wrinkle coupon, median strain–load slopes were ≈−14.5με/MPa ($\varepsilon_{002}$) and ≈−11.0με/MPa ($\varepsilon_{100}$); the small‐wrinkle coupon showed ≈−5.0 and −4.6με/MPa, respectively. Global d‐spacing trends gave a radial‐to‐axial contraction ratio of ≈2–2.5 in wrinkled specimens, while the pristine coupon remained near‐uniform.
iii) **Spatial localisation as a precursor to damage**. Microstrain maps revealed a compressive corridor flanking the wrinkle which was more pronounced in $\varepsilon_{002}$ than in $\varepsilon_{100}$. At 800N, XCT within these compressive strain zones confirmed delamination, matrix cracking and fibers kink‐banding, showing that strain mapping is capable of identifying failure‐prone regions several load steps ahead of catastrophic fracture.
iv) **Sub‐millistrain prediction via orientation‐aware modeling**. A lightweight predictor, anchored to load and incorporating spatial shape functions and signed misalignment Δ, reproduced measured fields and the corridor geometry across loads. Typical mean absolute errors of $\approx \left(0.5\text{--}0.9\right)\times 10^{-3}$ at 800N were obtained which are sufficient for triage and mapping of critical subregions.

This work employs a combination of in‐depth crystallographic characterisation and full‐field tomographic imaging to introduce a powerful new diagnostic capability. It demonstrates that radial microstrain gradients serve as a robust, non‐destructive indicator of wrinkle severity in carbon‐fibers laminates. This insight enables defect behavior to be embedded into full‐scale modeling, supporting performance‐based rejection criteria and targeted inspection in aerospace‐grade composites.

The significance of this approach lies in linking lattice‐level strain localisation with macroscopic damage mechanisms, offering a pathway to earlier intervention and more efficient inspection workflows. Rigour is demonstrated through automated data reduction, high‐fidelity orientation quantification, and validated predictive modeling across multiple specimens and load cases. Together, these elements establish a scalable, physics‐informed framework for studying damage evolution and guiding future research into fatigue, dwell loading, and multi‐scale inspection strategies.

The methodology is scalable, compatible with industrial NDE techniques, and suitable for deployment in production and maintenance settings. Future work could extend the protocol to fatigue and dwell loading to capture transitions from reversible lattice response to irreversible damage. Advances in orientation‐aware integration and uncertainty propagation would also improve strain quantification in highly misaligned regions. Fusion with techniques like phased‐array ultrasonics and thermography may help define modality‐agnostic thresholds based on $\varepsilon_{002}$ localisation, supporting cross‐validation and broader adoption.

## Conflict of Interest

The authors declare no conflict of interest.

## Author Contributions

H.M.L. contributed to data curation, formal analysis, investigation, methodology, software, validation, visualization, writing the original draft, and writing—review & editing; J.T. contributed to conceptualization, funding acquisition, project administration, resources, and supervision; I.D. contributed to funding acquisition, investigation, and resources; W.S. contributed to investigation and methodology; N.P. contributed to investigation and methodology; R.B. contributed to conceptualization, funding acquisition, project administration, supervision, and writing—review & editing; J.S. contributed to data curation, formal analysis, funding acquisition, investigation, methodology, project administration, software, supervision, validation, and writing—review & editing; and A.J.G.L. contributed to conceptualization, data curation, formal analysis, funding acquisition, investigation, methodology, project administration, resources, supervision, validation, writing the original draft, and writing—review & editing.

## Ethics

Ethical approval for the PhD associated with this project was secured from the University of Bath Ethics Panel. Patient/participant consent is not not applicable.

## Data Availability

The data that support the findings of this study are available from the corresponding author upon reasonable request.
